# Early‐life pathways to inequalities in child mental health: evidence from eight birth cohorts

**DOI:** 10.1111/jcpp.70207

**Published:** 2026-07-28

**Authors:** Demetris Avraam, Gabriella Melis, Tim Cadman, Jennie Carson, Marie Aline Charles, Yu Wei Chua, Ahmed Elhakeem, Joaquín Escribano, Veit Grote, Dariusz Gruszfeld, Kathrin Guerlich, Rae‐Chi Huang, Jennifer R. Harris, Jesus Ibarluzea, Jordi Julvez, Berthold Koletzko, Ashleigh Lin, Unai Martin, Rosemary McEachan, Maria Melchior, Phillip Melton, Johanna Thorbjornsrud Nader, Angela Pinot de Moira, Maja Popovic, Simona Porru, Lorenzo Richiardi, Blanca Sarzo, Daniela Schlüter, Jordi Sunyer, Morris Swertz, Elvira Verduci, John Wright, Annick Xhonneux, Tiffany Yang, Daniela Zugna, Anne‐Marie Nybo Andersen, Katrine Strandberg‐Larsen, David Taylor‐Robinson

**Affiliations:** ^1^ Department of Public Health University of Copenhagen Copenhagen Denmark; ^2^ Department of Public Health, Policy and Systems University of Liverpool Liverpool UK; ^3^ Department of Genetics, Genomics Coordination Center University Medical Center Groningen, University of Groningen Groningen The Netherlands; ^4^ Barcelona Institute for Global Health (ISGlobal) Barcelona Spain; ^5^ School of Population and Global Health The University of Western Australia Perth Australia; ^6^ French National Institute of Health and Medical Research (INSERM), French National Institute for Demographic Studies (INED), Joint Unit Elfe Aubervilliers France; ^7^ MRC Integrative Epidemiology Unit University of Bristol Bristol UK; ^8^ Department of Paediatrics, Sant Joan Reus Hospital University Rovira i Virgili, IISPV Reus Spain; ^9^ Division of Metabolic and Nutritional Medicine, Department of Paediatrics Dr. von Hauner Children's Hospital, LMU University Hospital Munich Germany; ^10^ German Center for Child and Adolescent Health, Site Munich Munich Germany; ^11^ Neonatal Department and Neonatal Intensive Care Unit Children's Memorial Health Institute Warsaw Poland; ^12^ Nutrition & Health Innovation Research Institute Edith Cowan University Perth Australia; ^13^ Centre for Fertility and Health Norwegian Institute of Public Health Oslo Norway; ^14^ Environmental Epidemiology and Child Development Unit Biogipuzkoa Health Research Institute Donostia‐San Sebastián Spain; ^15^ Spanish Consortium for Research and Public Health (CIBERESP) Instituto de Salud Carlos III Madrid Spain; ^16^ Clinical and Epidemiological Neuroscience (NeuroÈpia) Institut d'Investigació Sanitària Pere Virgili (IISPV) Reus Spain; ^17^ The Australian Research Centre in Sex, Health and Society La Trobe University Melbourne Vic. Australia; ^18^ Research Group in Social Determinants of Health and Demographic Change‐OPIK Department of Sociology and Social Work University of the Basque Country (UPV/EHU) Leioa (Biscay) Spain; ^19^ Bradford Institute for Health Research, Bradford Teaching Hospitals NHS Foundation Trust Bradford UK; ^20^ French National Institute of Health and Medical Research (INSERM) Institut Pierre Louis d'Epidémiologie et de Santé Publique (IPLESP), Epidémiologie Sociale, Santé Mentale Addictions (ESSMA) Paris France; ^21^ Menzies Institute for Medical Research, University of Tasmania Hobart Tas. Australia; ^22^ Department of Genetics and Bioinformatics, Division of Health Data and Digitalisation Norwegian Institute of Public Health Oslo Norway; ^23^ School of Public Health Imperial College London London UK; ^24^ Department of Medical Sciences University of Turin Turin Italy; ^25^ Department of Medicine, Faculty of Health Sciences Universitat Jaume I Castellón de la Plana Spain; ^26^ Epidemiology and Environmental Health Joint Research Unit FISABIO–Universitat Jaume I‐University of Valencia Valencia Spain; ^27^ Universitat Pompeu Fabra Barcelona Spain; ^28^ IMIM‐Parc Salut Mar Barcelona Catalonia Spain; ^29^ Pediatric Unit Fondazione IRCCS Ca' Granda Ospedale Maggiore Policlinico Milan Italy; ^30^ Groupe Santé CHC Liège Belgium

**Keywords:** Socioeconomic inequalities, externalising problems, internalising problems, multi‐cohort study, mediation analysis

## Abstract

**Background:**

Socioeconomic inequalities in children's emotional and behavioural problems are already present at school entry. We examined associations between early‐life socioeconomic conditions and children's externalising and internalising problems and assessed whether early‐life risk factors mediated these associations.

**Methods:**

We analysed harmonised individual participant data from eight birth cohorts within the EU Child Cohort Network. Maternal educational level during pregnancy was used as an indicator of early‐life socioeconomic conditions (SECs). Scores of children's externalising and internalising problems around school entry age were used as the outcome variables. Inequalities were quantified using the Slope Index of Inequality which captures the difference in prevalence of problems between children from highest and lowest SECs. Mediation analysis was used to assess the mediating role of five early‐life risk factors: maternal smoking during pregnancy, gestational age, small for gestational age, postpartum depression and breastfeeding.

**Results:**

Inequalities in externalising and internalising problems by SECs were consistent across cohorts: Children of mothers with low educational level had on average a 12 and 11 percentage point increased prevalence of externalising and internalising problems respectively. Early‐life factors explained around 8% (DNBC, Denmark) to 54% (INMA, Spain) of the inequality in externalising problems and 7% (ELFE, France) to 25% (ALSPAC, UK) in internalising problems.

**Conclusions:**

Children from lower SECs consistently had a higher prevalence of both externalising and internalising problems around school entry age. These inequalities were partly mediated by early‐life factors. Public health interventions in pregnancy and the preschool period are critical to address the early emergence of inequalities in children's mental health.

## Background

Child and adolescent mental health problems represent a significant global disease burden, affecting approximately 10%–20% of young people worldwide, with around half of all lifetime cases of mental health disorders emerging by the age of 14 (Kieling et al., 2011, 2024). There are clear social inequalities in child mental health problems based on childhood socioeconomic conditions (SECs), as commonly measured by parental education, income or occupation (Reiss, 2013).

Reducing inequalities in child mental health is a public health priority, yet the pathways that link social conditions to mental health outcomes, like emotional and behavioural problems in the early years, are unclear. Across various high‐income countries, marked social inequalities in internalising and externalising difficulties are evident, manifesting early in life and typically persisting throughout the childhood period (Cadman et al., 2024). However, few studies have explored the potential mediators of the social distribution and prevalence of mental health problems across countries or compared pathways to inequalities (Lai et al., 2020). Understanding these mechanisms is critical to guide public policy targeting improved child health and reduce inequalities.

Maternal education is a widely used indicator of children's socioeconomic conditions and may influence early‐life risk factors through multiple pathways (Pearce, Dundas, Whitehead, & Taylor‐Robinson, 2019). Lower maternal education may be associated with increased exposure to psychosocial stress, reduced access to healthcare and health information, and differences in health‐related behaviours during pregnancy and early childhood. For example, mothers with lower educational attainment are more likely to smoke during pregnancy (Kandel, Griesler, & Schaffran, 2009), experience postpartum depression (Wang et al., 2025), and have lower rates of breastfeeding (Neves et al., 2021). Socioeconomic disadvantage is also associated with adverse birth outcomes, including preterm birth and small for gestational age, potentially reflecting differences in maternal health, stress exposure and access to prenatal care (Ruiz et al., 2015). These early‐life risk factors may, in turn, contribute to inequalities in child mental health outcomes.

The pathways linking early‐life risk factors with child mental health may also differ for externalising and internalising problems. Externalising difficulties may be more strongly related to early neurodevelopmental and behavioural regulation processes influenced by prenatal exposures, caregiving environments and parental stress, whereas internalising problems may be more closely linked to emotional security, stress sensitivity, and parent–child relational processes. However, many early‐life risk factors are likely to influence both domains through overlapping biological and psychosocial mechanisms.

Previous studies in the United Kingdom (UK) have shown that common early‐years risk factors such as smoking in pregnancy and maternal perinatal mental health may explain a large proportion of the subsequent inequalities in child mental health. Perinatal factors alone were the most influential block of mediators, mediating over a third of the association of childhood socioeconomic conditions and mental health at age 14 years (Straatmann et al., 2018, 2019). Mediation by perinatal factors has been shown to be part of the pathways of inequalities in child mental health in both the UK and Denmark (Lai et al., 2020). Specific mediators are likely to be particularly important, including smoking in pregnancy, gestational age, birthweight, breastfeeding and maternal perinatal mental health.

It is not clear whether a consistent pattern of inequalities in child mental health exists across different countries, and how differently the early‐life risk factors mediate such inequalities in different contexts. For example, maternal perinatal mental health emerged as an important mediator of inequalities in child mental health in the UK, but less so in Denmark (Lai et al., 2020). Furthermore, differences might exist in the drivers of inequalities across types of child and adolescent mental health difficulties. Externalising problems involve disruptive behaviours (e.g. impulsivity, defiance), while internalising problems refer to the experience of emotions related to supressed behaviours (e.g. anxiety, sadness). Recent analysis of the UK Millennium Cohort Study shows the persistence of socioeconomic inequalities across both these measures, despite their different developmental trajectories (Chua, Schlüter, Pearce, Sharp, & Taylor‐Robinson, 2025).

Data from the EU Child Cohort Network (ECCN), established to bring together several pregnancy and childhood cohorts, provide a unique opportunity to answer this question (Jaddoe et al., 2020; Nader et al., 2023). In this study, we assess inequalities in externalising and internalising problems around school entry age; examine how early‐life risk factors mediate any inequalities; and assess how these relationships vary across contexts.

## Methods

### Study population

Eight cohorts within the ECCN had harmonised data on the variables of interest and agreed to participate. These cohorts are: the Avon Longitudinal Study of Parents and Children (ALSPAC, UK) (Boyd et al., 2013; Fraser et al., 2013), the Born in Bradford Study (BiB, UK) (McEachan et al., 2024; Wright et al., 2013), the European Childhood Obesity Project (CHOP, Belgium, Germany, Spain, Italy, Poland) (Koletzko et al., 2009), the Danish National Birth Cohort (DNBC, Denmark) (Olsen et al., 2001; Strandberg‐Larsen et al., 2025), the Étude Longitudinale Française depuis l'Enfance (ELFE, France) (Charles et al., 2020), the INfancia y Medio Ambiente Project (INMA, Spain) (Guxens et al., 2012), the Norwegian Mother, Father and Child Cohort (MoBa, Norway) (Magnus et al., 2016), and the Raine Study (The Raine Study, Australia) (Newnham, Evans, Michael, Stanley, & Landau, 1993). The study design as well as the characteristics for each cohort can be found in the respective cohort profile papers. All included cohorts were population‐based birth cohorts recruited from defined geographic areas and designed to be broadly representative of the general population, rather than high‐risk samples.

### Exposure

We used maternal educational level as the exposure variable, assessed during pregnancy or shortly after birth in each cohort, with recruitment spanning from 1989 to 2011. Maternal education was used as an indicator of socioeconomic position because it is a stable, widely recorded measure that strongly influences family resources, health behaviours, parenting practices and child development (Pearce et al., 2019). Maternal educational level was harmonised across cohorts using the International Standard Classification of Education 97 (ISCED‐97), a UNESCO‐developed framework that enables harmonisation of educational attainment across countries by mapping national education systems onto a common set of internationally comparable categories (UNESCO United Nations Educational, Scientific and Cultural Organization, 2003). The harmonised variable consisted of three categories: *Low* (No education to lower secondary; ISCED‐97 categories 0–2), *Medium* (Upper and post‐secondary; ISCED‐97 categories 3–4), *High* (Degree and above; ISCED‐97 categories 5–6). We calculated a scaled maternal education by ranking the three maternal educational levels from the lowest to the highest and allocating a score (ranging from 0 to 1) that equals the midpoint of the category's range in the cumulative distribution. For example, if 24% of the mothers in a cohort had low education, they would be allocated a score of 0.12 (0.24/2), and if 42% of mothers had medium and 34% high education, they would be allocated a score of 0.45 (0.24 + 0.42/2) and 0.83 (0.24 + 0.42 + 0.34/2) respectively. The scaled maternal education was used as a continuous exposure variable for the calculation of the Slope Index of Inequality (SII) and in mediation analysis (Regidor, 2004).

### Outcomes

Parent‐reported scores of children's internalising and externalising problems were used as the outcome variables. Different instruments were used by the cohorts to quantify behavioural problems (Nader et al., 2023): the Strengths and Difficulties Questionnaire (SDQ) (Goodman, 1997), the Child Behaviour Checklist (CBCL) (Achenbach & Edelbrock, 1991) and the Youth Self‐Report (YSR) (Ebesutani, Bernstein, Martinez, Chorpita, & Weisz, 2011) (see Box S1 in Appendix S1). To enable comparison across cohorts, percentile scores of externalising/internalising problems were used, indicating each child's relative position within their group (cohort and age specific) as measured by a standardised instrument. A percentile score has been assigned to each child (at each age) using the values of their externalising/internalising raw scores. The distribution of measurement age is shown in Figure S1. We used the percentile score for every child closest to school entry age (median across all cohorts: 5.6 years, interquartile range: 4.6–5.8). Measuring the outcome close to the age where the mediators are measured reduces bias due to unaccounted mediator‐outcome confounding (Vo, Superchi, Boutron, & Vansteelandt, 2020). The selected percentile scores were dichotomised based on the 90th percentile value of each cohort to identify the 10% of the children with the highest percentile scores, indicating more severe emotional and behavioural problems (Guerlich et al., 2024). The derived binary indicators, one for externalising and one for internalising problems, were used as the outcome variables in the analysis.

### Confounders

Maternal age at birth is considered a confounder as outlined in our directed acyclic graph (Figure 1). Other potential confounders such as maternal pre‐pregnancy mental health, ethnicity and parity were not considered due to the unavailability of harmonised data across all participating cohorts.

**Figure 1:**
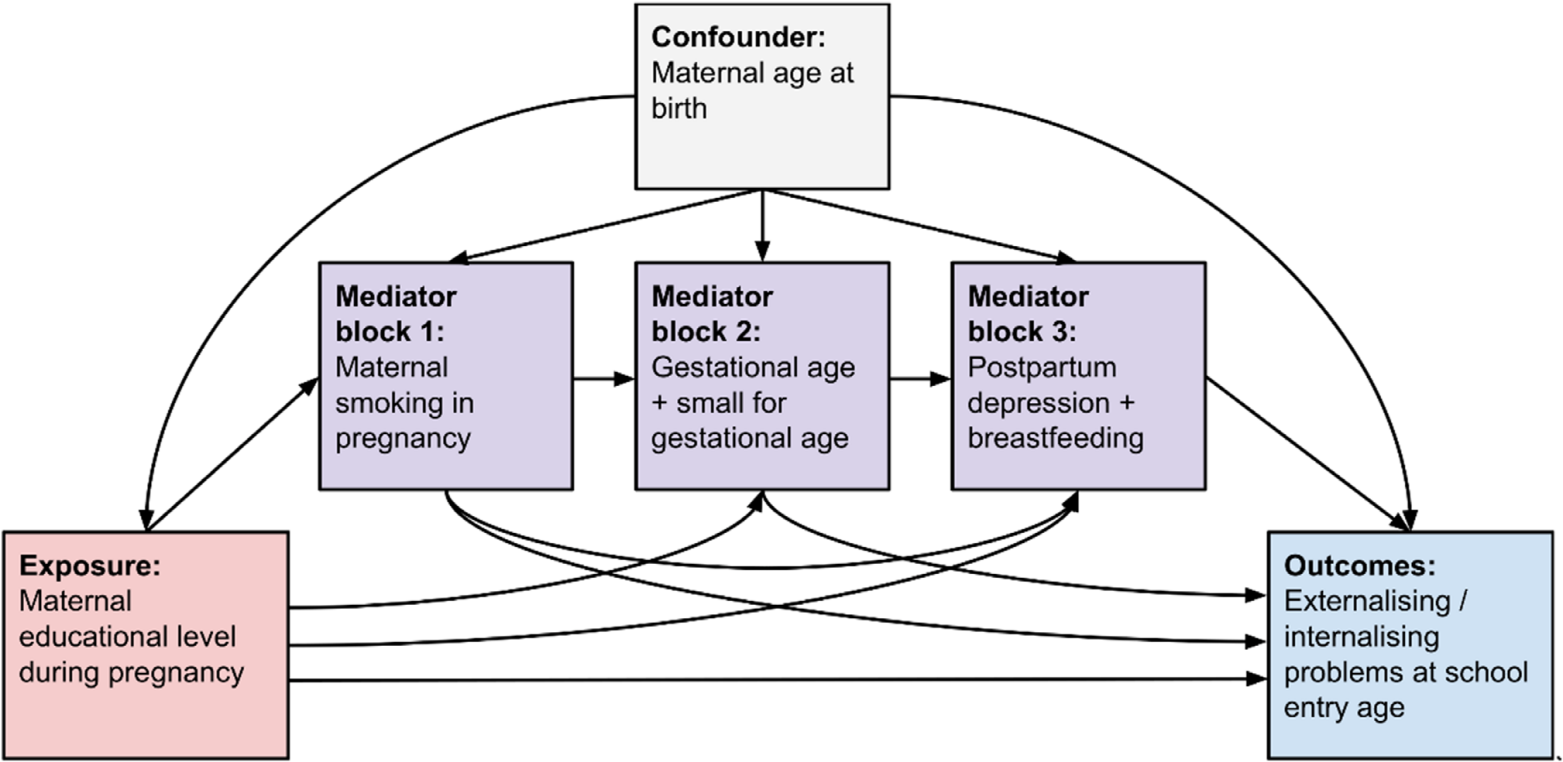
Directed acyclic graph representing the current study

### Mediators

We identified several early‐life risk factors (Lai et al., 2020; Nakamura, François, & Lepeule, 2021; Straatmann et al., 2019) that may mediate the social gradient in child mental health and we assessed five of them based on the availability of harmonised data. The following were included: (a) maternal smoking in pregnancy (yes/no), (b) gestational age (in days), (c) small for gestational age (SGA) (yes/no), (d) postpartum depression (yes/no), and (e) breastfeeding (yes/no) (see Box S2 in Appendix S1). The five potential mediators were assessed in three blocks ordered by temporality and added in the analysis sequentially: pregnancy period (block 1: maternal smoking in pregnancy), at birth (block 2: gestational age and SGA), postnatal period (block 3: postpartum depression and breastfeeding).

### Statistical analysis

The analysis progressed in two steps. First, the inequalities in the distribution of the mediators and outcomes in each of the cohorts were assessed. We estimated the prevalence of externalising and internalising problems at each level of maternal education and calculated the SII. An overall SII was estimated as a weighted average of the cohort‐specific SIIs where the weights corresponded to the complete case sample sizes of the cohorts. The complete cases were defined by the data records without any missing values for all variables used in the analysis (Figure 2).

**Figure 2:**
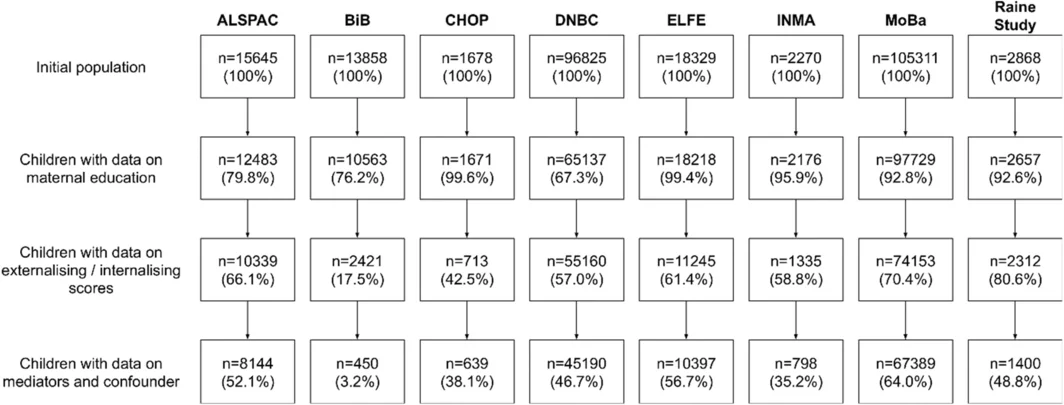
Flow chart showing how the final size (and the percentage relative to the initial sample size) of the complete cases is derived in each cohort based on the availability of measurements for the exposure, outcome, mediators, and confounder. Note that the data of externalising/internalising problems in BiB were collected in a subsample and not on the full cohort, and data of postpartum depression were collected only for the BiB1000 sub‐study (Wright et al., 2016) which included 1763 mother–child dyads, resulting in a big reduction in our complete cases sample size

Second, a counterfactual mediation approach to estimate the extent to which early‐life factors mediated the association between maternal education and child mental health outcomes was used. This approach allows decomposition of total effects into direct and indirect effects, providing insight into potential mechanisms linking socioeconomic conditions and child mental health. The proportion of the inequality mediated was also calculated, which quantifies how much of the observed relationship can be attributed to these indirect pathways distinct from the direct effect of socioeconomic conditions (Vanderweele, 2015). While this approach strengthens causal interpretation, it relies on assumptions including no unmeasured confounding of exposure–mediator and mediator–outcome relationships, which may not be fully testable in observational studies. Poisson regression models were used to estimate the Risk Ratio (RR) and 95% Confidence Interval (CI) for the natural direct effect (NDE), natural indirect effect (NIE) and total effect (TE). In the analysis, the natural direct effect estimates the change in outcome when maternal education is varied (low vs high) while mediators are fixed to the average levels observed in the high‐education group. The natural indirect effect estimates the change in risk when maternal education is fixed at the high level but mediator values are assigned according to scenarios expected under low maternal education. The proportion mediated (PM) was calculated when the natural direct and indirect effects were in the same direction (i.e. either both RRs of NDE and NIE greater than 1 or both lower than 1) using the formula:

$$\frac{RR_{\mathrm{NDE}}\left(RR_{\mathrm{NIE}}-1\right)}{\left(RR_{\mathrm{NDE}}\times RR_{\mathrm{NIE}}-1\right)}.$$

Separate mediation analyses were undertaken in different cohorts. We compare the results, rather than seeking to undertake a pooled (or meta‐) analysis, given the different prevalence of the mediators and possibly different mediating mechanisms in different settings and over time.

All statistical analyses were conducted using DataSHIELD, which is an R‐based tool allowing federated remote analysis of individual‐level data without the need of data sharing or transfer (Avraam et al., 2025; Gaye et al., 2014; Wilson et al., 2017). We mainly used the DataSHIELD *dsBase* package (version 6.3.0) for data manipulation, descriptive and exploratory statistics and the *dsMediation* package (version 0.0.3) for mediation analysis. For the mediation analysis we specifically used DataSHIELD wrapper functions from the *medflex* R package (version 0.6‐10) that permits an imputation‐based approach and fits natural effect models, a class of counterfactual models to directly parameterise the path‐specific effects of interest, in the presence of single or multiple mediators (Steen, Loeys, Moerkerke, & Vansteelandt, 2017).

### Robustness tests

Several sensitivity analyses to assess the robustness of the main results were performed, assessing assumptions about: the impact of missing data; the selection of the age interval at which the outcome variables are measured; any effect modification by child sex; and using equivalised household income as an alternative measure of socioeconomic conditions (see Appendix S2 for further details).

## Results

### Mother and child characteristics

The initial number of children in the participating cohorts varied between 1678 (CHOP) and 105,311 (MoBa); however, their number ranged between 450 (BiB) and 67,389 (MoBa) in the complete cases analysis samples (Figure 2). Mothers and children characteristics in the complete cases sample of each cohort are shown in Table 1, while the characteristics in the initial samples of the cohorts are shown in Table S1.

**Table 1:** Mothers and children's characteristics in the complete cases sample of each cohort

	ALSPAC	BiB	CHOP	DNBC	ELFE	INMA	MoBa	Raine Study
Birth years	1991–1992	2007–2011	2002–2004	1996–2002	2011	1997–2008	1999–2008	1989–1992
*Study population*								
*n*	8,144	450	639	45,190	10,397	798	67,389	1,400
*Sex*								
Male (*n*, %)	4,161 (51.1%)	214 (47.6%)	292 (45.7%)	22,345 (49.4%)	5,327 (51.2%)	393 (49.2%)	34,511 (51.2%)	712 (50.9%)
Female (*n*, %)	3,983 (48.9%)	236 (52.4%)	347 (54.3%)	22,845 (50.6%)	5,070 (48.8%)	405 (50.8%)	32,878 (48.8%)	688 (49.1%)
*Maternal age at birth, in years*								
Mean (*SD*)	28.9 (4.6)	28.0 (5.6)	30.7 (4.7)	30.2 (4.2)	30.9 (4.6)	32.3 (3.8)	30.4 (4.4)	28.7 (5.7)
*Maternal education level*								
Low (*n*, %)	1,182 (14.5%)	266 (59.1%)	121 (18.9%)	5,963 (13.2%)	435 (4.2%)	133 (16.7%)	1,213 (1.8%)	672 (48.0%)
Medium (*n*, %)	5,697 (70.0%)	57 (12.7%)	325 (50.9%)	16,828 (37.2%)	2,875 (27.7%)	325 (40.7%)	20,412 (30.3%)	429 (30.6%)
High (*n*, %)	1,265 (15.5%)	127 (28.2%)	193 (30.2%)	22,399 (49.6%)	7,087 (68.2%)	340 (42.6%)	45,764 (67.9%)	299 (21.4%)
*Maternal smoking in pregnancy*								
No (*n*, %)	6,279 (77.1%)	409 (90.9%)	455 (71.2%)	34,262 (75.8%)	8,676 (83.4%)	606 (75.9%)	53,160 (78.9%)	1,083 (77.4%)
Yes (*n*, %)	1865 (22.9%)	41 (9.1%)	184 (28.8%)	10,928 (24.2%)	1721 (16.6%)	192 (24.1%)	14,229 (21.1%)	317 (22.8%)
*Gestational age, in days*								
Mean (*SD*)	279.8 (11.7)	276.0 (12.3)	282.8 (8.4)	280.0 (11.4)	277.4 (9.9)	279.4 (9.4)	279.2 (12.5)	274.3 (14.8)
*Birth weight, in kg*								
Mean (*SD*)	3.4 (0.5)	3.1 (0.6)	3.3 (0.3)	3.6 (0.5)	3.3 (0.5)	3.3 (0.4)	3.6 (0.6)	3.3 (0.6)
Birth weight for gestational age								
Small (*n*, %)	591 (7.3%)	72 (16.0%)	57 (8.9%)	1,524 (3.4%)	700 (6.7%)	66 (8.3%)	1880 (2.8%)	75 (5.4%)
Normal or large (*n*, %)	7,553 (92.7%)	378 (84.0%)	582 (91.1%)	43,666 (96.6%)	9,697 (93.3%)	732 (91.7%)	65,509 (97.2%)	1,325 (94.6%)
*Postpartum depression*								
No (*n*, %)	7,495 (92.0%)	352 (78.2%)	561 (87.8%)	38,246 (84.6%)	9,509 (91.5%)	708 (88.7%)	60,640 (90.0%)	1,271 (90.8%)
Yes (*n*, %)	649 (8.0%)	98 (21.8%)	78 (12.2%)	6,944 (15.4%)	888 (8.5%)	90 (11.3%)	6,749 (10.0%)	129 (9.2%)
*Child ever breast fed*								
No (*n*, %)	1898 (23.3%)	69 (15.3%)	171 (26.8%)	767 (1.7%)	2,476 (23.8%)	58 (7.3%)	4,319 (6.4%)	118 (8.4%)
Yes (*n*, %)	6,246 (76.7%)	381 (84.7%)	468 (73.2%)	44,423 (98.3%)	7,921 (76.2%)	740 (92.7%)	63,070 (93.6%)	1,282 (91.6%)
*Externalising problems*								
Score < the 90th percentile^a^ (*n*, %)	7,160 (87.9%)	403 (89.6%)	575 (90.0%)	40,636 (89.9%)	9,186 (88.4%)	709 (88.8%)	60,164 (89.3%)	1,240 (88.6%)
Score ≥ the 90th percentile^a^ (*n*, %)	984 (12.1%)	47 (10.4%)	64 (10.0%)	4,554 (10.1%)	1,211 (11.6%)	89 (11.2%)	7,225 (10.7%)	160 (11.4%)
*Internalising problems*								
Score < the 90th percentile^a^ (*n*, %)	7,262 (89.2%)	399 (88.7%)	575 (90.0%)	40,387 (89.4%)	9,197 (88.5%)	715 (89.6%)	58,655 (87.0%)	1,255 (89.6%)
Score ≥ the 90th percentile^a^ (*n*, %)	882 (10.8%)	51 (11.3%)	64 (10.0%)	4,803 (10.6%)	1,200 (11.5%)	83 (10.4%)	8,734 (13.0%)	145 (10.4%)

^a^ Using the 90th percentile values of the complete cases sample of each cohort.

### Inequalities in early‐life risk factors

The associations between maternal education and potential mediators and confounders are shown in Figure 3. In all cohorts, mothers with lower education were more likely to smoke during pregnancy, have postpartum depression, and give birth at a younger age. Children of mothers with lower education were more likely to have a shorter gestational age, be smaller‐for‐gestational age, and less likely to be breastfed. The SIIs of these relationships and their weighted average across all cohorts are shown in Figure S2. Cross‐tabulations between maternal education and the examined early‐life factors are shown in Table S2.

**Figure 3:**
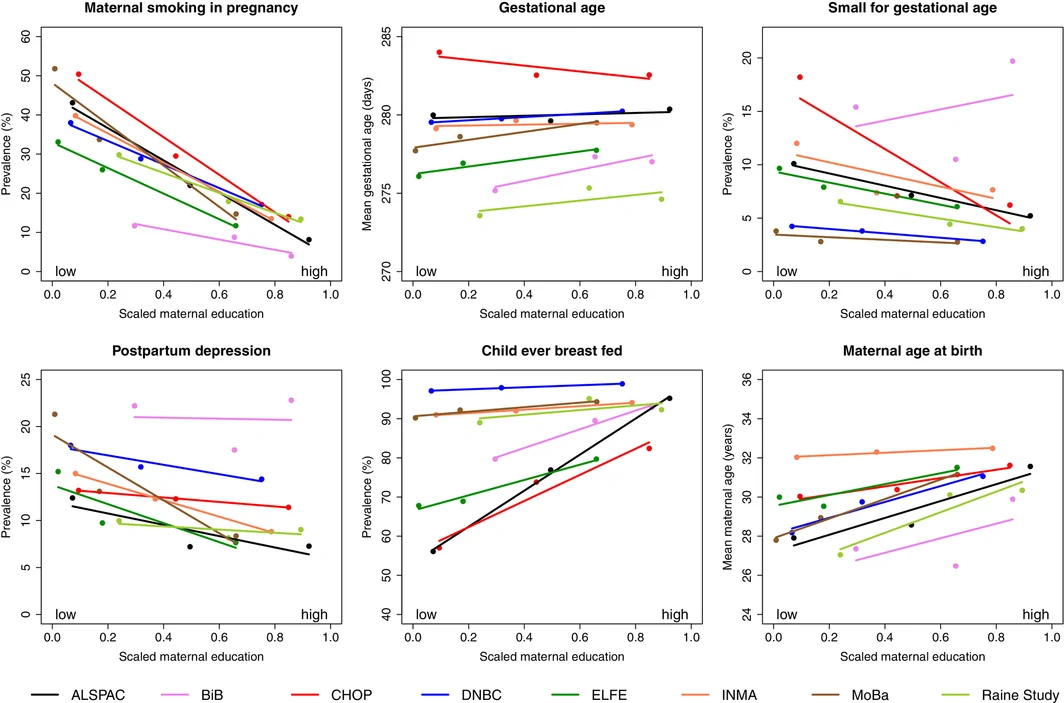
Prevalence or mean of early‐life factors at each level of scaled maternal education. The slope of each trendline defines the Slope Index of Inequality. For example, the SII for maternal smoking in ALSPAC cohort is −41.1, interpreted as the percentage points difference in the prevalences of maternal smoking in pregnancy between mothers of highest (3.7% is the prevalence at scaled maternal education = 1) and lowest (44.8% is the prevalence at scaled maternal education = 0) values on the scaled maternal educational hierarchy

### Inequalities in externalising and internalising problems

The associations between scaled maternal education and the prevalence of externalising and internalising problems are shown in Figure 4. On average, children of mothers with low educational level had a 12.0 and 10.9 percentage point increased prevalence of externalising and internalising problems respectively, compared to children of mothers with high‐educational level. The cohort‐specific SIIs ranged between −6.1 (95% CI: −9.1 to −3.0, Raine Study) and −20.7 (95% CI: −22.0 to −19.5, CHOP) for externalising problems. The SIIs for internalising problems ranged between −5.6 (95% CI: −8.9 to −2.3, ALSPAC) and −18.8 (95% CI: −33.8 to −3.7, BiB), other than the Raine Study which does not show any inequality (SII: −0.2, 95% CI: −6.6 to 6.2).

**Figure 4:**
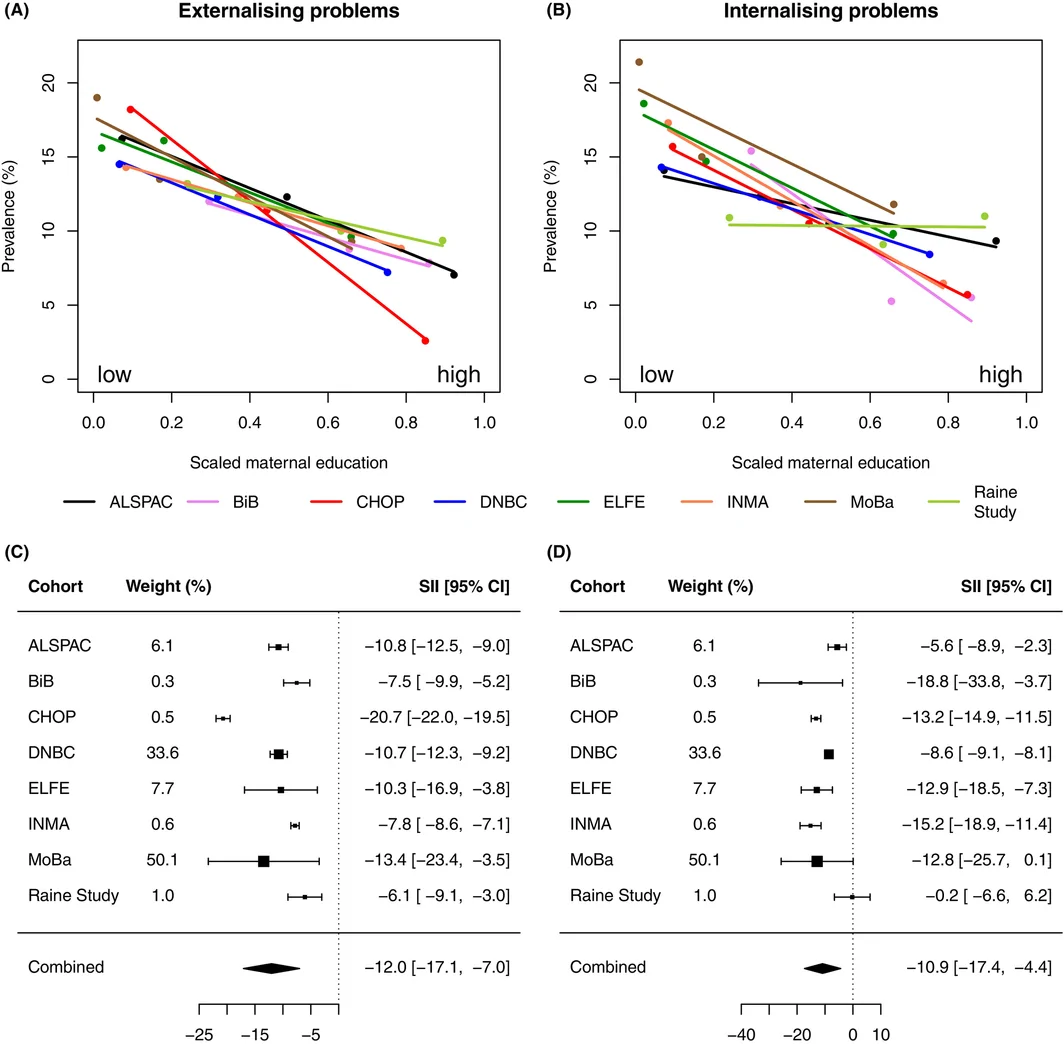
Prevalence of externalising (A) and internalising problems (B) at each level of scaled maternal education. The slope of each trendline defines the Slope Index of Inequality. The forest plots summarise the SIIs and their 95% confidence intervals for externalising (C) and internalising (D) problems. The combined SII is calculated as the weighted average of the cohort‐specific SIIs where the weights are defined by the size of the complete case sample in each cohort

### Mediation effects of early‐life risk factors in externalising and internalising problems

The results from mediation analysis are shown in Table 2. The total effect of maternal education on children's externalising problems (the risk ratio comparing children with the highest and lowest SECs) ranged between 0.12 (95% CI: 0.05 to 0.27, CHOP) and 0.76 (95% CI: 0.42 to 1.37, Raine Study), indicating a lower risk of problems in children of higher educated mothers. The weighted average of the total effects of SECs on externalising problems across all cohorts was 0.43 (95% CI: 0.41 to 0.46) (Figure S3). The natural direct effect for externalising problems ranged between 0.12 (95% CI: 0.05 to 0.28, CHOP) and 0.86 (95% CI: 0.48 to 1.55, Raine Study) while the natural indirect effect ranged between 0.66 (95% CI: 0.51 to 0.86, INMA) and 1.04 (95% CI: 0.80 to 1.35, BiB), in the model with all blocks of mediators. In BiB and CHOP there was no evidence of an indirect effect. In the other cohorts, the proportion mediated by maternal smoking in pregnancy ranged between 5% (ELFE) and 45% (Raine Study). Adding gestational age and small for gestational age into the mediation model did not substantially alter the proportions mediated which ranged between 6% (ELFE) and 40% (Raine Study). Adding postpartum depression and breastfeeding increased the proportion mediated to between 8% (DNBC) and 54% (INMA).

**Table 2:** Estimates from causal mediation analysis for the associations between maternal education and externalising problems (A) and between maternal education and internalising problems (B)

Mediator	Estimate	ALSPAC	BiB	CHOP	DNBC	ELFE	INMA	MoBa	Raine Study
A. Externalising problems									
	Total effect	0.46 [0.36, 0.59]	0.51 [0.15, 1.70]	0.12 [0.05, 0.27]	0.38 [0.34, 0.43]	0.39 [0.31, 0.48]	0.51 [0.25, 1.05]	0.48 [0.44, 0.52]	0.76 [0.42, 1.37]
Maternal smoking in pregnancy	Natural direct effect	0.54 [0.42, 0.70]	0.54 [0.16, 1.78]	0.11 [0.05, 0.26]	0.42 [0.38, 0.47]	0.42 [0.34, 0.52]	0.67 [0.32, 1.42]	0.52 [0.47, 0.57]	0.87 [0.48, 1.57]
	Natural indirect effect	0.85 [0.80, 0.90]	0.95 [0.84, 1.07]	1.03 [0.84, 1.28]	0.91 [0.89, 0.93]	0.92 [0.88, 0.96]	0.76 [0.62, 0.94]	0.92 [0.90, 0.94]	0.87 [0.79, 0.97]
	Proportion mediated	14.8%	5.7%	–	6.2%	5.4%	32.8%	8.3%	44.7%
+ Gestational age + small for gestational age	Natural direct effect	0.54 [0.42, 0.70]	0.52 [0.16, 1.71]	0.12 [0.05, 0.28]	0.42 [0.38, 0.47]	0.42 [0.34, 0.53]	0.68 [0.32, 1.45]	0.52 [0.48, 0.57]	0.85 [0.47, 1.54]
	Natural indirect effect	0.85 [0.80, 0.90]	0.98 [0.81, 1.20]	0.99 [0.78, 1.26]	0.90 [0.88, 0.92]	0.91 [0.87, 0.95]	0.75 [0.59, 0.94]	0.91 [0.89, 0.93]	0.89 [0.80, 0.98]
	Proportion mediated	15.2%	1.8%	0.2%	6.7%	6.4%	35.1%	8.8%	39.9%
+ Postpartum depression + breastfeeding	Natural direct effect	0.59 [0.45, 0.76]	0.49 [0.15, 1.61]	0.12 [0.05, 0.28]	0.43 [0.39, 0.48]	0.44 [0.36, 0.55]	0.77 [0.37, 1.61]	0.55 [0.50, 0.61]	0.86 [0.48, 1.55]
	Natural indirect effect	0.79 [0.72, 0.86]	1.04 [0.80, 1.35]	0.99 [0.75, 1.31]	0.88 [0.86, 0.90]	0.87 [0.83, 0.92]	0.66 [0.51, 0.86]	0.86 [0.84, 0.88]	0.88 [0.79, 0.98]
	Proportion mediated	23.1%	–	0.1%	8.1%	9.1%	53.5%	14.4%	41.6%
B. Internalising problems									
	Total effect	0.68 [0.51, 0.90]	0.15 [0.04, 0.61]	0.27 [0.11, 0.66]	0.53 [0.48, 0.58]	0.47 [0.38, 0.59]	0.25 [0.12, 0.55]	0.63 [0.58, 0.69]	1.14 [0.63, 2.08]
Maternal smoking in pregnancy	Natural direct effect	0.73 [0.55, 0.99]	0.13 [0.03, 0.55]	0.30 [0.12, 0.79]	0.56 [0.50, 0.62]	0.47 [0.38, 0.59]	0.36 [0.16, 0.81]	0.65 [0.60, 0.71]	1.30 [0.70, 2.39]
	Natural indirect effect	0.92 [0.87, 0.98]	1.10 [0.99, 1.23]	0.87 [0.68, 1.12]	0.94 [0.93, 0.96]	0.99 [0.95, 1.04]	0.70 [0.55, 0.87]	0.97 [0.95, 0.99]	0.88 [0.80, 0.98]
	Proportion mediated	18.1%	–	5.2%	6.7%	0.5%	14.7%	5.6%	–
+ Gestational age + small for gestational age	Natural direct effect	0.74 [0.55, 0.99]	0.13 [0.03, 0.51]	0.32 [0.12, 0.83]	0.56 [0.51, 0.63]	0.49 [0.39, 0.61]	0.36 [0.16, 0.80]	0.66 [0.60, 0.72]	1.32 [0.72, 2.42]
	Natural indirect effect	0.92 [0.87, 0.97]	1.18 [1.00, 1.40]	0.84 [0.64, 1.11]	0.94 [0.92, 0.95]	0.97 [0.93, 1.02]	0.70 [0.56, 0.89]	0.96 [0.94, 0.98]	0.87 [0.78, 0.96]
	Proportion mediated	18.6%	–	6.9%	7.6%	2.6%	14.1%	6.6%	–
+ Postpartum depression + breastfeeding	Natural direct effect	0.76 [0.57, 1.02]	0.11 [0.03, 0.45]	0.36 [0.14, 0.94]	0.58 [0.52, 0.64]	0.51 [0.41, 0.64]	0.38 [0.17, 0.85]	0.70 [0.65, 0.77]	1.32 [0.73, 2.41]
	Natural indirect effect	0.89 [0.82, 0.98]	1.32 [0.99, 1.75]	0.75 [0.55, 1.01]	0.91 [0.89, 0.93]	0.93 [0.88, 0.98]	0.67 [0.53, 0.85]	0.90 [0.88, 0.92]	0.86 [0.77, 0.96]
	Proportion mediated	25.0%	–	12.6%	11.2%	6.9%	16.8%	19.1%	–

The estimates include the total effect, the natural direct effect, the natural indirect effect, and the proportion mediated by each block of mediators added sequentially in the model. The total, natural direct and natural indirect effects are given as risk ratios with 95% confidence intervals. The proportion mediated is not calculated when the natural direct and natural indirect effects have opposite directions.

The total effects of scaled maternal education on internalising problems ranged between 0.15 (95% CI: 0.04 to 0.61, BiB) and 0.68 (95% CI: 0.51 to 0.90, ALSPAC) for the seven cohorts that had a negative SII. As observed earlier in Figure 4, in the Raine Study the SII for internalising problems was null and for this cohort the total effect was greater than 1 (RR: 1.14, 95% CI: 0.62 to 2.10). The weighted average of the total effects of maternal education on internalising problems across all cohorts was 0.58 (95% CI: 0.54 to 0.62) (Figure S3). In BiB and the Raine Study, the direct and indirect effects had opposite directions, while in CHOP and ELFE there was no evidence of an indirect effect. Within the cohorts with evidence of an indirect effect, the proportion mediated by maternal smoking in pregnancy ranged between 6% (MoBa) and 18% (ALSPAC). When adding gestational age and small for gestational age in the model, the proportion mediated slightly increased and ranged between 7% (MoBa) and 19% (ALSPAC). Further adding postpartum depression and breastfeeding in the model, the proportion mediated increased and ranged between 7% (ELFE) and 25% (ALSPAC). Regression estimates for all pairwise relationships between exposure, outcome and mediators are shown in Figures S4–S6.

### Robustness tests

The results were robust to our sensitivity analyses (see Appendix S2). We found similar SIIs as those found in the main analysis, when re‐scaling maternal education and dichotomising the outcome percentile scores based on their univariate non‐missing values (Figures S7–S9). We also found similar SIIs when imputing missing values in the mediators and confounders (Tables S3, S4). Narrowing the age interval from 0–20 to 0–10 years, for which the outcome variables are selected also provided similar SIIs as the main analysis (Figures S10–S13, Tables S5, S6). Furthermore, based on *p*‐values from Wald tests, we concluded that child's sex is not an effect modifier (Table S7, Figures S14–S19). This is in line with previous studies which found that the effect of socioeconomic inequalities on mental health did not vary by child sex in the UK (Chua et al., 2025). Finally, we found consistent directions of the inequalities when using household income as an alternative indicator of SECs (Figures S20–S23, Table S8); however, the magnitude of the inequalities was smaller than those derived using maternal education, as the two measures capture different dimensions of SECs (Galobardes, Shaw, Lawlor, Lynch, & Davey Smith, 2006).

## Discussion

Using data from eight birth cohorts across different countries participating in the ECCN, this study demonstrates three key findings. First, child mental health inequalities were highly consistent across cohorts: children growing up in socioeconomically disadvantaged circumstances had a higher risk of developing both emotional and behavioural problems at school entry age compared with peers from more advantaged families. For example, children of mothers with a low educational level had a 12.0 and 10.9 percentage point increased prevalence of externalising and internalising problems, respectively, compared with children of mothers with a high‐educational level. Second, early‐life risk factors related to maternal and newborn health and well‐being explained a substantial share of these inequalities, with similar indirect effects observed across cohorts, but the proportion of inequalities mediated varied by context, ranging from around 8% in Denmark (DNBC) to 54% in Spain (INMA) for externalising problems, and from around 7% in France (ELFE) to 25% in the UK (ALSPAC) for internalising problems. Third, the extent of mediation differed between outcomes: early‐life factors explained a larger share of socioeconomic inequalities in externalising problems than in internalising problems, with the weighted average of the total effect across all cohorts being RR 0.43 (95% CI: 0.41–0.46) for externalising and RR 0.58 (95% CI: 0.54–0.62) for internalising problems.

Variation in findings across cohorts may reflect contextual differences, including recruitment period, population characteristics, policy environments, and measurement approaches. Cohorts spanned different time periods, during which social policies, maternal employment patterns, and healthcare provision may have changed, potentially influencing the social patterning of early‐life exposures and child mental health outcomes. In addition, cohorts differed in geographic coverage, ranging from national to regional populations, which may affect socioeconomic distributions and access to services. Differences in measurement instruments and levels of missing data may also contribute to variation in observed inequalities and mediation effects. These contextual differences highlight the importance of interpreting cross‐cohort findings within their broader social and policy environments.

### Potential biological mechanisms

Early‐life exposures can influence neurodevelopment through mechanisms including impaired foetal growth, altered neuroendocrine regulation, and epigenetic modifications (Arango et al., 2018). Prenatal exposure to maternal smoking, for instance, has been linked to epigenetic changes and alterations in the structure and function of the developing foetal brain (Nakamura et al., 2021). Adverse birth outcomes, such as preterm birth or low birth weight, are markers of suboptimal foetal development and are associated with increased risk for neurodevelopmental disadvantages. For example, one study examined the association between being small for gestational age and reduced white matter integrity in newborns and increased prevalence of psychiatric symptoms in adulthood (Eikenes et al., 2012).

Postnatal factors also play a critical role. Maternal postpartum psychiatric disorders, particularly depression, can disrupt mother–infant bond, a key determinant of early emotional and cognitive development. Exposure to postpartum depression has been associated with increased risks of emotional and behavioural problems, developmental delays, feeding and sleep difficulties, and challenges in social functioning (Bernard‐Bonnin, Society Canadian Paediatric, & Committee Mental Health and Developmental Disabilities, 2004). Breastfeeding supports maternal–infant emotional connection through the release of oxytocin and contributes to the regulation of the infant's stress response. Breast milk also contains beneficial nutrients, such as long‐chain polyunsaturated fatty acids, which are important for neurocognitive and brain development (Bugaeva et al., 2023). Although the individual effect sizes of these factors are often small, their impact may be amplified when they co‐occur and cluster with other adversities. This cumulative exposure can increase susceptibility to later mental health problems and contribute to the broader effects of social disadvantage.

### Comparisons with previous studies

Previous research has consistently shown that children from socioeconomically disadvantaged families are at greater risk of mental health difficulties, and that early‐life factors explain part of these inequalities. For example, studies using the UK Millennium Cohort Study and the Danish National Birth Cohort found that perinatal factors and maternal mental health accounted for around one‐third of inequalities in child and adolescent mental health (Lai et al., 2020; Straatmann et al., 2019). Our findings are consistent with this pattern, but by using a block‐wise approach across eight cohorts, we are able to highlight which early‐life factors are most influential. In particular, maternal smoking in pregnancy emerged as a key mediator. However, as we did not account for maternal mental health during pregnancy, smoking may also act as a marker of the broader psychosocial context.

Cross‐cohort comparisons have previously suggested international variation in the extent of inequalities and their persistence over time (Cadman et al., 2024). Our study extends this work by focusing specifically on outcomes at around school age when they first present and demonstrating that early‐life factors explain a larger share of socioeconomic inequalities in externalising than in internalising problems. We also show that the contribution of early‐life factors estimated by the indirect effects is similar across countries, but the proportion of inequality they explain varies by context. Together, this points to both common pathways across settings and important differences in the magnitude of mediation by early‐life factors.

Cross‐country differences in mediation effects may reflect variation in social policies, healthcare systems, and levels of socioeconomic inequality. Countries with more comprehensive welfare provision, universal access to healthcare, and stronger parental support policies may reduce socioeconomic inequalities in early‐life conditions, including maternal health behaviours, birth outcomes, and early caregiving practices. These contexts may also influence the social patterning of maternal mental health, with socioeconomic differences in perinatal mental health varying across countries depending on the availability of support services and broader social protections. In contrast, in settings with greater socioeconomic inequalities or differences in service provision, early‐life factors may play a larger role in shaping inequalities in child mental health. These findings highlight the importance of broader structural and policy contexts in shaping early‐life pathways to socioeconomic inequalities in child mental health.

### Strengths and limitations

This study has several notable strengths. We used harmonised data from eight large birth cohorts in the ECCN spanning Australia and multiple European countries, enhancing generalisability across high‐income settings and enabling meaningful cross‐cohort comparisons. The consistent harmonisation of exposures, mediators and outcomes allowed us to apply a counterfactual mediation framework to investigate pathways from socioeconomic disadvantage to child emotional and behavioural problems. Importantly, the use of the DataSHIELD infrastructure enabled advanced analyses to be conducted across cohorts without the transfer of individual‐level data, demonstrating the feasibility of secure, federated approaches for life‐course research (Avraam et al., 2025).

At the same time, several limitations must be acknowledged. Our mediation analysis assumes no unmeasured mediator–outcome confounding, yet information on maternal mental health during pregnancy and other potential confounders was unavailable. As with many cross‐cohort analyses, harmonisation of variables across studies required prioritising comparability, which limited the inclusion of some potentially important factors. For example, maternal smoking during pregnancy may reflect broader psychosocial adversity, including maternal mental health, which was not consistently available across cohorts. As a result, some mediators may represent proxies for more complex underlying processes related to maternal and newborn health and well‐being, rather than isolated causal factors. These limitations highlight the potential for residual confounding and suggest that findings should be interpreted cautiously when considering policy implications. Nevertheless, the consistent identification of early‐life factors across multiple cohorts supports the importance of early‐life environments in shaping socioeconomic inequalities in child mental health. Reliance on parent‐reported measures, differences in outcome measurement instruments and assessment ages, and unavoidable missing data are further limitations, though sensitivity analyses suggest these did not materially alter results. We also lacked data on some relevant mediators (e.g. paternal mental health, domestic violence, migration status), and could not calculate confidence intervals for the proportion mediated due to software constraints.

Future research should extend this work by examining both the biological mechanisms and the psychosocial context surrounding maternal smoking in pregnancy, as well as incorporating richer measures of maternal mental health. Expanding analyses to low‐resource settings and more ethnically diverse populations is also critical, given the intersections between socioeconomic disadvantage, ethnicity and maternal health risks. Continued development of federated platforms such as DataSHIELD (Avraam et al., 2025) and collaborative initiatives like the EU Child Cohort Network (Jaddoe et al., 2020) will be essential to address these questions with greater data richness, methodological rigour and cross‐cohort scope.

## Conclusion

Using harmonised data from eight birth cohorts across 10 high‐income countries, this study shows large socioeconomic inequalities in child emotional and behavioural problems that are evident at school entry age and consistent across settings. A substantial share of these inequalities can be traced to early‐life factors linked to maternal and newborn health and well‐being. In the context of rising child mental health problems, these findings highlight the need for earlier intervention to improve mental health across the life course. Strengthening perinatal and early childhood support, reducing exposures such as maternal smoking, and addressing the broader social determinants of health could help narrow mental health inequalities before school entry and reduce mental health inequalities across the life course.

## Ethical considerations

This study was approved by the executive board of the EU Child Cohort Network on 16 June 2020 (ECCNLC202039) and separately by each cohort included in the study. Ethical approval for the original data collection in each cohort was granted by the respective local ethics boards (Table S10), and no additional ethical approval was needed for this secondary data analysis. All participants provided written informed consent (Table S9).

**Key points**

**What is known?**

- A limited evidence base suggests some mediation effect of early‐life factors on the association between socioeconomic circumstances and childhood mental health problems including emotional and behavioural symptoms but most research assessed early‐life factors individually.
- Previous studies were conducted using data from the UK, France, Denmark and the United States, but only one study included a cross‐national comparison between the UK and Denmark.

**What is new?**

- This study provides the most comprehensive evidence on the mediating role of five early‐life factors considered together in the relationship between socioeconomic inequalities and early childhood internalising and externalising problems across eight birth cohort studies from the UK, Belgium, Germany, Spain, Italy, Poland, France, Norway, Denmark and Australia.
- While there is a consistent difference in the prevalence of internalising and externalising problems between the most and least disadvantaged socioeconomic groups, the proportion of inequality explained by early‐life factors varies by context, ranging from up to one‐quarter for internalising difficulties to as much as one‐half for externalising difficulties.

**What is relevant?**

- Public health interventions that address inequalities in maternal and newborn health and well‐being are important for reducing childhood inequalities in externalising and internalising behaviours.

## Supporting information


**Appendix S1.** Supplementary information for the main analysis.
**Box S1.** Instruments used to assess children's emotional and behavioural problems.
**Figure S1.** Frequency of available externalising (red colour) and internalising (green colour) scores across ages per cohort.
**Box S2.** Information of data collected on potential mediators.
**Table S1.** Mothers and children characteristics in the initial sample of each cohort.
**Figure S2.** Slope Index of Inequalities between scaled maternal education and early‐life risk factors as calculated based on trendlines in Figure 3.
**Table S2.** Cross‐tabulations between maternal education and the mediators and confounder on the complete cases sample.
**Figure S3.** Estimates from the relationships between the exposure and the outcomes (total effect).
**Figure S4.** Estimates of the relationships between the exposure and the mediators.
**Figure S5.** Estimates of the relationships between the mediators and externalising problems.
**Figure S6.** Estimates of the relationships between the mediators and internalising problems.
**Appendix S2.** Supplementary information for the sensitivity analyses.
**Figure S7.** Prevalence or mean of early‐life factors at each level of scaled maternal education using their pairwise complete cases.
**Figure S8.** Slope Index of Inequalities between scaled maternal education and early‐life risk factors as calculated based on trendlines in Figure S7.
**Figure S9.** Prevalence of externalising (A) and internalising problems (B) at each level of scaled maternal education using the maternal education and the outcome variables scaled and dichotomised respectively using the initial sample sizes.
**Table S3.** Mothers and children characteristics in the five imputed samples per cohort.
**Table S4.** Slope index of inequality and 95% CI between scaled maternal education and each mediator and confounder and between scaled maternal education and externalising and internalising problems for each imputed dataset.
**Figure S10.** Frequency of externalising (red colour) and internalising (green colour) scores at the child ages when the outcome variables are selected for the main analysis.
**Table S5.** Complete cases sample size.
**Table S6.** Child age in years (median and interquartile range (IQR)) when the outcome variables are measured.
**Figure S11.** Prevalence or mean of early‐life factors at each level of scaled maternal education using their complete cases derived from the narrowed age interval used for the selection of the outcome variable.
**Figure S12.** Slope Index of Inequalities between scaled maternal education and early‐life risk factors as calculated based on trendlines in Figure S11.
**Figure S13.** Prevalence of externalising (A) and internalising problems (B) at each level of scaled maternal education using the maternal education and the outcome variables scaled and dichotomised respectively using the sample sizes of the complete cases derived from the narrowed age interval used for the selection of the outcome variable.
**Table S7.** Wald test *p*‐values of the estimates of the interaction term examining whether child's sex is an effect modifier for the relationship between maternal education and externalising or internalising problems.
**Figure S14.** Prevalence or mean of early‐life factors at each level of scaled maternal education using the complete cases samples for male children.
**Figure S15.** Slope Index of Inequalities between scaled maternal education and early‐life risk factors as calculated based on trendlines in Figure S14.
**Figure S16.** Prevalence of externalising (A) and internalising problems (B) at each level of scaled maternal education using the maternal education and the outcome variables scaled and dichotomised respectively using the sample sizes of the complete cases samples for male children.
**Figure S17.** Prevalence or mean of early‐life factors at each level of scaled maternal education using the complete cases samples for female children.
**Figure S18.** Slope Index of Inequalities between scaled maternal education and early‐life risk factors as calculated based on trendlines in Figure S17.
**Figure S19.** Prevalence of externalising (A) and internalising problems (B) at each level of scaled maternal education using the maternal education and the outcome variables scaled and dichotomised respectively using the sample sizes of the complete cases samples for female children.
**Figure S20.** Flow chart showing how the final size (and the percentage relative to the initial sample) of the complete cases is derived in each cohort based on the availability of measurements for the exposure (household income), outcome and mediators/confounders.
**Table S8.** Baseline characteristics of the participants in the complete cases sample among children with information on household income.
**Figure S21.** Prevalence or mean of early‐life factors at each level of scaled household income used as an alternative exposure measure of SECs.
**Figure S22.** Slope Index of Inequalities between scaled household income and early‐life risk factors as calculated based on trendlines in Figure S21.
**Figure S23.** Prevalence of externalising (A) and internalising problems (B) at each level of scaled household income quintile.
**Appendix S3.** Supplementary information for cohort‐specific statements.
**Box S3.** Description of the participating cohorts.
**Table S9.** Informed consent statements.
**Table S10.** Ethics approval statements.
**Table S11.** Funding and acknowledgement statements.

## Data Availability

This study used harmonised birth cohort data from the EU Child Cohort Network (ECCN), established under the Horizon2020 LifeCycle project. Variable details and harmonisation procedures are available in the ECCN data catalogue. Data are not publicly available; access requires a proposal to the ECCN committee and subsequent approval by individual cohorts, after which accredited researchers are granted remote access via DataSHIELD, ensuring no individual‐level data leave host sites. For data access enquiries, contact the coordinating centre (eu.child.cohort@erasmusmc.nl); for cohort‐specific data sharing policies, contact the respective cohorts.
