# Supplementary material for: Early‐life pathways to inequalities in child mental health: evidence from eight birth cohorts
Source: J Child Psychol Psychiatry. 2026 Jul 28;67(12):2084–98. doi: 10.1111/jcpp.70207 (PMC13745791; doi:10.1111/jcpp.70207)
Supplement: Supplementary file 1 [file JCPP-67-2084-s001.docx]

**Early-life pathways to inequalities in child mental health: Evidence from eight birth cohorts**

**Supporting Information**

**Appendix S1: Supplementary information for the main analysis**

**Box S1: Instruments used to assess children’s emotional and behavioural problems:**

The Strengths and Difficulties Questionnaire (SDQ) (1) was used in ALSPAC (at 4, 7, 8, 9, 11, 13, and 16 years), BiB (at 4 years), CHOP (at 5.5 and 11 years), DNBC (at 7, 11 and 18 years), ELFE (at 5-6 years) and INMA (at 10-12 years) cohorts. The Child Behaviour Checklist (CBCL) (2) was used in CHOP (at 8 years), INMA (at 7-9 years), MoBa and the Raine Study (at 2, 5, 8, 10 years). The Youth Self-Report (YSR) (3) was used in the Raine Study at 13 and 16 years. Parent‐report questionnaires were used for all cohorts and ages except the third data collection wave at age 18 years for DNBC where self‐report of CBCL was used and the use of YSR for the Raine Study at ages 13 and 16.

The SDQ contains 25 items measured on a 3‐point Likert scale (“not true”, “somewhat true”, “certainly true”) containing five subscales: emotional problems, conduct problems, hyperactivity, peer problems, and pro‐social behaviour. Internalising scores are calculated by combining the emotional problems and peer problems subscales, while externalising scores are calculated by combining the conduct problems and hyperactivity subscales. The CBCL for school ages (6-18 years) consists of 113 items while the CBCL for preschool ages (1.5-5 years) contains 99 items rated on a 3‐point Likert scale (“not true”, “sometimes true”, “often true”). The CBCL contains eight subscales: delinquent behaviour, aggressive behaviour, withdrawn, somatic complaints, anxious/depressed, social problems, thought problems and attention problems. Internalising scores are calculated by combining the withdrawn, somatic complaints, and anxious/depressed subscales, while externalising scores are calculated by combining the delinquent and aggressive behaviours subscales. The YSR is a 112-item self-report designed for children and adolescents (ages 11-17), that assesses behavioural competency and behavioural problems and parallels the CBCL. Behaviours in YSR are rated on a 3-point Likert scale (“not true”, “somewhat or sometimes true”, “very true or often true”) and providing scores for seven subscales: anxious/depressed, withdrawn/depressed, somatic complains, social problems, thought problems, rule-breaking behaviour, and aggressive behaviour. Internalising and externalising scores are calculated by combining the same subscales in YSR as in CBCL.

An externalising or internalising score is calculated as the average of raw scores of the relevant subscales based on completed items in the questionnaires. Cases with more than 25% of the items comprising a subscale incomplete were considered as missing.

**Figure S1:** Frequency of available externalising (red colour) and internalising (green colour) scores across ages per cohort.

**Box S2: Information of data collected on potential mediators:**

a) Maternal smoking in pregnancy was self-reported by mothers and defined as any maternal smoking during pregnancy.
b) Gestational age at birth was obtained using each cohort’s best clinical judgement: a) using the last menstrual period if the difference from ultrasound measurement was less than seven days or if ultrasound was not available, b) using ultrasound if the difference between last menstrual period and ultrasound was more than seven days or if last menstrual period was not available, c) using maternal report if none of them was available.
c) Small for gestational age was calculated by converting the weight of the child at birth (in grams) to z-scores, adjusted by gestational age and sex, using international standards from the INTERGROWTH-21^st^ project (4). Infants whose birth weight z-score was below -1.28 (which is equivalent to the 10^th^ centile of the z-scores distribution) were classified as small for gestational age (SGA) (5). Children with gestational age below 24 and above 43 weeks were excluded.
d) Postpartum depression was defined using a self-reported scale (e.g. the Edinburgh Postnatal Depression Scale (EPDS)) or was clinically diagnosed and categorised as a yes vs. no indicators. For ALSPAC, cases were defined as EPDS>12 measured at approximately 12 weeks postpartum. For BiB, General Health Questionnaire items were scored and summed to give a total score with a possible range of 0-84. Mothers with a cut-off >=23 were considered to have postpartum depression. For CHOP, an EPDS score >=13 at any timepoint (2, 3 or 6 months after birth) was defined as postpartum depression. For DNBC, a maternal distress variable was used from questionnaire data and a diagnosis for women with very severe depression within the first year of child's life from registry data. A ROC (Receiver Operating Characteristic) curve was fitted to the data (outcome: registry-based depression, exposure: maternal postpartum distress variable) and the cut-off (for the maternal distress variable) with the maximised combined sensitivity and specificity was chosen. For ELFE, cases were defined as EPDS>12 measured between 6 and 8 weeks postpartum. For MoBa, postpartum depression was defined through a self-reported questionnaire at 6 months after birth. For the Raine Study, cases were defined from the EPDS.
e) Breastfeeding was self-reported by mothers and defined by whether the child was ever breastfed (yes/no).

**Table S1:** Mothers and children characteristics in the initial sample of each cohort.

|  | **ALSPAC** | **BiB** | **CHOP** | **DNBC** | **ELFE** | **INMA** | **MoBa** | **Raine Study** |
| --- | --- | --- | --- | --- | --- | --- | --- | --- |
| **Study population** |  |  |  |  |  |  |  |  |
| n | 15645 | 13858 | 1678 | 96825 | 18329 | 2270 | 105311 | 2868 |
| **Sex** |  |  |  |  |  |  |  |  |
| Male (n, %) | 7706 (51.2%) | 7153 (51.6%) | 850  (50.7%) | 49644 (51.3%) | 9318 (51.4%) | 1099 (51.5%) | 53865 (51.2%) | 1454 (50.7%) |
| Female (n, %) | 7355 (48.8%) | 6704 (48.4%) | 828  (49.3%) | 47181 (48.7%) | 8811 (48.6%) | 1037 (48.5%) | 51251 (48.8%) | 1414 (49.3%) |
| Missing (n, %) | 584 (3.7%) | 1  (0%) | 0  (0%) | 0  (0%) | 200 (1.1%) | 134 (5.9%) | 195 (0.2%) | 0  (0%) |
| **Maternal age at birth, in years** |  |  |  |  |  |  |  |  |
| Mean (SD) | 28.4 (4.8) | 27.5 (5.6) | 29.8 (5.1) | 30.0 (4.3) | 30.2 (5.1) | 31.7 (4.3) | 30.2 (4.6) | 27.6 (5.9) |
| Missing (n, %) | 4199 (26.8%) | 0  (0%) | 9  (0.5%) | 3  (0%) | 196  (1.1%) | 223  (9.8%) | 169  (0.2%) | 77  (2.7%) |
| **Maternal educational level** |  |  |  |  |  |  |  |  |
| Low (n, %) | 2526 (20.2%) | 5986 (56.7%) | 423 (25.3%) | 9346  (14.3%) | 1555 (8.5%) | 575 (26.4%) | 2766 (2.8%) | 1448 (54.5%) |
| Medium (n, %) | 8348 (66.9%) | 1657 (15.7%) | 838 (50.1%) | 24621 (37.8%) | 6344 (34.8%) | 891 (40.9%) | 32204 (33.0%) | 694 (26.1%) |
| High (n, %) | 1609 (12.9%) | 2920 (27.6%) | 410 (24.5%) | 31170 (47.9%) | 10319 (56.6%) | 710 (32.6%) | 62759 (64.2%) | 515 (19.4%) |
| Missing (n, %) | 3162 (20.2%) | 3295 (23.8%) | 7  (0.4%) | 31688 (32.7%) | 111  (0.6%) | 94  (4.1%) | 7582 (7.2%) | 211  (7.4%) |
| **Household income** |  |  |  |  |  |  |  |  |
| First quintile (n, %) | 2158 (20%) | 2171 (20%) | 308  (20.2%) | 16368  (20%) | 2708 (20%) | 387  (20%) | 17893 (19.8%) | NA |
| Second quintile (n, %) | 2166 (20%) | 2171 (20%) | 304  (20%) | 16470  (20%) | 2709 (20%) | 387  (20%) | 18120 (20%) | NA |
| Third quintile (n, %) | 2152 (19.9%) | 2171 (20%) | 304  (20%) | 16370 (20%) | 2709 (20%) | 387  (20%) | 17960 (19.8%) | NA |
| Fourth quintile (n, %) | 2187 (20.3%) | 2171 (20%) | 304  (20%) | 16417 (20%) | 2709 (20%) | 387  (20%) | 17332 (19.2%) | NA |
| Fifth quintile (n, %) | 2126 (19.7%) | 2171 (20%) | 303 (19.9%) | 16417 (20%) | 2709 (20%) | 386  (20%) | 19179 (21.2%) | NA |
| Missing (n, %) | 4856 (31.0%) | 3003 (21.7%) | 155  (9.2%) | 14783 (15.3%) | 4785 (26.1%) | 336 (14.8%) | 14827 (14.1%) | NA |
| **Maternal smoking in pregnancy** |  |  |  |  |  |  |  |  |
| No (n, %) | 8203 (69.1%) | 9568 (83.5%) | 1092 (65.2%) | 69077 (73.4%) | 14374 (79.9%) | 1432 (67.8%) | 79957 (76.6%) | 1982 (70.7%) |
| Yes (n, %) | 3663 (30.9%) | 1885 (16.5%) | 583 (34.8%) | 25021 (26.6%) | 3624 (20.1%) | 679 (32.2%) | 24433 (23.4%) | 822 (29.3%) |
| Missing (n, %) | 3779 (24.2%) | 2405 (17.4%) | 3  (0.2%) | 2727  (2.8%) | 331  (1.8%) | 159  (7.0%) | 921  (0.9%) | 64  (2.2%) |
| **Gestational age, in days** |  |  |  |  |  |  |  |  |
| Mean (SD) | 271.9 (38.0) | 276.0 (13.5) | 282.6  (8.5) | 278.4 (14.2) | 276.8 (10.4) | 278.9 (11.9) | 278.4 (14.1) | 273.5 (16.9) |
| Missing (n, %) | 1064 (6.8%) | 332  (2.4%) | 15  (0.9%) | 4  (0%) | 391  (2.1%) | 144  (6.3%) | 2684 (2.5%) | 119  (4.1%) |
| **Birth weight, in kg** |  |  |  |  |  |  |  |  |
| Mean (SD) | 3.4 (0.6) | 3.2 (0.6) | 3.3 (0.3) | 3.5 (0.6) | 3.3 (0.5) | 3.3 (0.5) | 3.6 (0.6) | 3.3 (0.6) |
| Missing (n, %) | 1782 (11.4%) | 334  (2.4%) | 5  (0.3%) | 683  (0.7%) | 503  (2.7%) | 150  (6.6%) | 18085 (17.2%) | 80  (2.8%) |
| **Birth weight for gestational age** |  |  |  |  |  |  |  |  |
| Small (n, %) | 1165 (8.4%) | 1757 (13.0%) | 173 (10.4%) | 4115 (4.3%) | 1359 (7.7%) | 242 (11.5%) | 2706 (3.1%) | 157 (5.9%) |
| Normal or Large (n, %) | 12643 (91.6%) | 11756  (87.0%) | 1489 (89.6%) | 91647 (95.7%) | 16354 (92.3%) | 1860  (88.5%) | 84342 (96.9%) | 2517 (94.1%) |
| Missing (n, %) | 1837 (11.7%) | 345  (2.5%) | 16 (1.0%) | 1063 (1.1%) | 616  (3.4%) | 168  (7.4%) | 18263 (17.3%) | 194  (6.8%) |
| **Postpartum depression** |  |  |  |  |  |  |  |  |
| No (n, %) | 10340 (91.3%) | 1048 (79.7%) | 901 (84.5%) | 58815 (83.9%) | 14308 (90.8%) | 968 (87.8%) | 78084 (89.3%) | 1487 (90.5%) |
| Yes (n, %) | 986 (8.7%) | 267  (20.3%) | 165  (15.5%) | 11297 (16.1%) | 1443 (9.2%) | 135  (12.2%) | 9343 (10.7%) | 156  (9.5%) |
| Missing (n, %) | 4319 (27.6%) | 12543  (90.5%) | 612 (36.5%) | 26713 (27.6%) | 2578 (14.1%) | 1167 (51.4%) | 17884 (17.0%) | 1225 (42.7%) |
| **Child ever breast fed** |  |  |  |  |  |  |  |  |
| No (n, %) | 2881 (26.3%) | 896  (21.2%) | 507 (30.2%) | 1283  (1.8%) | 5471 (33.0%) | 224  (11.4%) | 5899 (6.6%) | 258  (10.7%) |
| Yes (n, %) | 8082 (73.7%) | 3332 (78.8%) | 1170 (69.8%) | 68972 (98.2%) | 11111 (67.0%) | 1744 (88.6%) | 83418 (93.4%) | 2159 (89.3%) |
| Missing (n, %) | 4682 (29.9%) | 9630 (69.5%) | 1  (0.1%) | 26570 (27.4%) | 1747 (9.5%) | 302 (13.3%) | 15994 (15.2%) | 451 (15.7%) |
| **Externalising problems** |  |  |  |  |  |  |  |  |
| Scores < the 90^th^ percentile^*^ (n, %) | 10077 (89.6%) | 2270 (87.5%) | 637 (89.0%) | 68405 (89.6%) | 9942 (88.4%) | 1212 (89.6%) | 70307 (89.1%) | 2229 (89.4%) |
| Scores ≥ the 90^th^ percentile^*^ (n, %) | 1164 (10.4%) | 324  (12.5%) | 79  (11.0%) | 7934 (10.4%) | 1306 (11.6%) | 140  (10.4%) | 8610 (10.9%) | 264  (10.6%) |
| Missing (n, %) | 4404 (28.1%) | 11264 (81.3%) | 962 (57.3%) | 20486 (21.2%) | 7081 (38.6%) | 918 (40.4%) | 26394 (25.1%) | 375 (13.1%) |
| **Internalising problems** |  |  |  |  |  |  |  |  |
| Scores < the 90^th^ percentile^*^ (n, %) | 9962 (88.6%) | 2287 (88.2%) | 638 (89.2%) | 68148 (89.3%) | 9932 (88.3%) | 1215 (89.9%) | 68574 (86.8%) | 2241 (89.9%) |
| Scores ≥ the 90^th^ percentile^*^ (n, %) | 1279 (11.4%) | 307 (11.8%) | 77 (10.8%) | 8204 (10.7%) | 1315 (11.7%) | 137 (10.1%) | 10413 (13.2%) | 252  (10.1%) |
| Missing (n, %) | 4404 (28.1%) | 11264 (81.3%) | 963 (57.4%) | 20473 (21.1%) | 7082 (38.6%) | 918 (40.4%) | 26324 (25.0%) | 375 (13.1%) |

^*^ Using the 90^th^ percentile values of the initial sample of each cohort

Note that the percentages of non-missing values sum up to 100% (with some deviation due to rounding) while the percentage of missing values is based on the whole sample size of each cohort.

**Figure S2:** Slope Index of Inequalities between scaled maternal education and early-life risk factors as calculated based on trendlines in Figure 3. The combined SII in each panel is calculated as the weighted average of the cohort-specific SIIs. The weights are defined by the size of the complete cases sample of each cohort.

**Table S2:** Cross tabulations between maternal education and the mediators and confounder on the complete cases sample

|  | **ALSPAC** |  |  | **BiB** |  |  | **CHOP** |  |  | **DNBC** |  |  |
| --- | --- | --- | --- | --- | --- | --- | --- | --- | --- | --- | --- | --- |
| **Maternal educational level** | **Low** | **Medium** | **High** | **Low** | **Medium** | **High** | **Low** | **Medium** | **High** | **Low** | **Medium** | **High** |
| **Maternal smoking in pregnancy** |  |  |  |  |  |  |  |  |  |  |  |  |
| Yes % | 43.1 | 22.0 | 8.1 | 11.7 | 8.8 | 3.9 | 50.4 | 29.5 | 14.0 | 38.0 | 28.8 | 17.1 |
| **Gestational age, in days** |  |  |  |  |  |  |  |  |  |  |  |  |
| Mean | 280.0 | 279.6 | 280.4 | 275.2 | 277.3 | 277.0 | 284.0 | 282.5 | 282.5 | 279.5 | 279.8 | 280.2 |
| **Small for gestational age** |  |  |  |  |  |  |  |  |  |  |  |  |
| Yes % | 10.1 | 7.1 | 5.2 | 15.4 | 10.5 | 19.7 | 18.2 | 7.1 | 6.2 | 4.2 | 3.8 | 2.8 |
| **Postpartum depression** |  |  |  |  |  |  |  |  |  |  |  |  |
| Yes % | 12.4 | 7.2 | 7.3 | 22.2 | 17.5 | 22.8 | 13.2 | 12.3 | 11.4 | 18.0 | 15.7 | 14.4 |
| **Child ever breast fed** |  |  |  |  |  |  |  |  |  |  |  |  |
| Yes % | 56.1 | 76.9 | 95.2 | 79.7 | 89.5 | 92.9 | 57.0 | 73.8 | 82.4 | 97.1 | 97.9 | 98.9 |
| **Maternal age at birth** |  |  |  |  |  |  |  |  |  |  |  |  |
| Mean | 27.9 | 28.6 | 31.6 | 27.4 | 26.5 | 29.9 | 30.0 | 30.4 | 31.6 | 28.2 | 29.8 | 31.1 |

**Table S2 (continued):** Cross tabulations between maternal education and the mediators and confounder on the complete cases sample

|  | **ELFE** |  |  | **INMA** |  |  | **MoBa** |  |  | **Raine Study** | | |
| --- | --- | --- | --- | --- | --- | --- | --- | --- | --- | --- | --- | --- |
| **Maternal educational level** | **Low** | **Medium** | **High** | **Low** | **Medium** | **High** | **Low** | **Medium** | **High** | **Low** | **Medium** | **High** |
| **Maternal smoking in pregnancy** |  |  |  |  |  |  |  |  |  |  |  |  |
| Yes % | 33.1 | 26.0 | 11.7 | 39.8 | 28.6 | 13.5 | 51.8 | 33.7 | 14.7 | 29.8 | 17.9 | 13.4 |
| **Gestational age, in days** |  |  |  |  |  |  |  |  |  |  |  |  |
| Mean | 276.1 | 276.9 | 277.7 | 279.1 | 279.6 | 279.4 | 277.7 | 278.6 | 279.5 | 273.6 | 275.3 | 274.6 |
| **Small for gestational age** |  |  |  |  |  |  |  |  |  |  |  |  |
| Yes % | 9.7 | 7.9 | 6.1 | 12.0 | 7.4 | 7.7 | 3.8 | 2.8 | 2.8 | 6.6 | 4.4 | 4.0 |
| **Postpartum depression** |  |  |  |  |  |  |  |  |  |  |  |  |
| Yes % | 15.2 | 9.7 | 7.7 | 15.0 | 12.3 | 8.8 | 21.3 | 13.11 | 8.4 | 10.0 | 8.2 | 9.0 |
| **Child ever breast fed** |  |  |  |  |  |  |  |  |  |  |  |  |
| Yes % | 67.8 | 68.9 | 79.7 | 91.0 | 92.0 | 94.1 | 90.2 | 92.2 | 94.3 | 89.0 | 95.1 | 92.3 |
| **Maternal age at birth** |  |  |  |  |  |  |  |  |  |  |  |  |
| Mean | 30.0 | 29.5 | 31.5 | 32.0 | 32.3 | 32.5 | 27.8 | 28.9 | 31.1 | 27.1 | 30.1 | 30.3 |

**Figure S3: Estimates from the relationships between the exposure and the outcomes (total effect).** Forest plots showing the cohort-specific estimates of the exposure variable (scaled maternal education). We ran Poisson regressions adjusted by maternal age at birth. The combined estimate is calculated as the weighted average of the cohort-specific estimates. The weights are defined by the size of the complete cases sample of each cohort.

**Figure S4: Estimates of the relationships between the exposure and the mediators.** Forest plots showing the cohort-specific estimates of the exposure variable (scaled maternal education). We ran Poisson regressions for the binary mediators and linear regression for the continuous mediator (gestational age). All models adjusted by maternal age at birth. The combined estimate is calculated as the weighted average of the cohort-specific estimates. The weights are defined by the size of the complete cases sample of each cohort.

**Figure S5: Estimates of the relationships between the mediators and externalising problems.** Forest plots showing the cohort-specific estimates of the mediators from Poisson regressions. Each model is adjusted by maternal age at birth and by the mediators of the same or previous blocks. The combined estimate is calculated as the weighted average of the cohort-specific estimates. The weights are defined by the size of the complete cases sample of each cohort.

**Figure S6: Estimates of the relationships between the mediators and internalising problems.** Forest plots showing the cohort-specific estimates of the mediators from Poisson regressions. Each model is adjusted by maternal age at birth and by the mediators of the same or previous blocks. The combined estimate is calculated as the weighted average of the cohort-specific estimates. The weights are defined by the size of the complete cases sample of each cohort.

**Appendix S2: Supplementary information for the sensitivity analyses**

***Robustness test: The impact of missing data***

To examine the impact of excluding children with missing values at any of the variables of interest on our results, we conducted two sensitivity analyses. First, we re-calculated the Slope Index of Inequality (SII) of the considered relationships using the non-missing values of each variable separately. To do that we (i) scaled maternal education based on the proportions of mothers at each educational level on the initial sample of each cohort; (ii) dichotomised the percentile scores of externalising/internalising problems using the 90^th^ percentile of the variables in the initial sample of each cohort; and (iii) calculated the prevalence of each binary mediator (i.e. maternal smoking, SGA, postpartum depression and breast feeding) and the mean of each continuous mediator/confounder (i.e. gestational age and maternal age at birth) by each category of maternal education using the pairwise complete cases from the initial samples. Second, we imputed missing values in the mediators/confounders and re-calculated the SIIs. We used a DataSHIELD wrapper function from the mice R package (version 3.16.0) that allows multiple imputations with chained equations (6). We created 5 imputed datasets in each cohort. We then calculated the SIIs from each imputed dataset and finally pooled them using the Rubin’s rule.

***Calculate the slope index of inequalities using the initial sample***

When we re-calculated the SII of the considered relationships using the non-missing values of each variable separately instead of using the values in the final complete cases sample, we did not observe any significant differences. The weighted averages of the SIIs across all cohorts in the main analysis were -42.3, 1.8, -2.2, -11.7, 8.4, 4.4 (see Figure S2) and in the sensitivity analysis were -44, 2.1, -2.3, -12.2, 12.2, 5 (see Figures S7 and S8) for maternal smoking, gestational age, small for gestational age, postpartum depression, breastfeeding and maternal age at birth respectively. All small differences where within the CIs of the corresponding SIIs. Similarly, we observed negligible differences in the slopes between scaled maternal education and externalising and internalising problems. The combined SIIs in the sensitivity analysis were -12.2 and -11.1 (see Figure S9) compared to -12.0 and -10.9 (see Figure 4) in the main analysis for externalising and internalising problems respectively.

**Figure S7:** Prevalence or mean of early-life factors at each level of scaled maternal education using their pairwise complete cases. The slope of each trendline defines the Slope Index of Inequality.

**Figure S8:** Slope Index of Inequalities between scaled maternal education and early-life risk factors as calculated based on trendlines in Figure S7. The combined SII in each panel is calculated as the weighted average of the cohort-specific SIIs. The weights are defined by each pairwise complete cases sample size of each cohort.

**Figure S9:** Prevalence of externalising (A) and internalising problems (B) at each level of scaled maternal education using the maternal education and the outcome variables scaled and dichotomised respectively using the initial sample sizes. The slope of each trendline defines the Slope Index of Inequality. The forest plots summarise the SIIs and their 95% confidence intervals for externalising (C) and internalising (D) problems. The combined SII is calculated as the weighted average of the cohort-specific SIIs where the weights are defined by each pairwise complete cases sample size of each cohort.

***Impute missing values in mediators and confounder***

When we imputed missing values in the mediators/confounders we also did not observe any significant differences with the results from the main analysis. The imputations gave similar statistics of the baseline characteristics (compare Table 1 with Table S3). The SIIs obtained from imputations were also similar both at variable and cohort level (see the pooled values calculated by Rubin’s Rule, in Table S4 and compare them with the values of SIIs in Figures 4 and S2) and at combined level (the weighted averages of SIIs in the imputed data across all cohorts were equivalent with the weighted averages of SIIs derived from the main analysis).

**Table S3:** Mothers and children characteristics in the five imputed samples per cohort.

|  | **ALSPAC** | **BiB** | **CHOP** | **DNBC** | **ELFE** | **INMA** | **MoBa** | **Raine Study** |
| --- | --- | --- | --- | --- | --- | --- | --- | --- |
| **Study population** |  |  |  |  |  |  |  |  |
| n | 10339 | 2421 | 713 | 55160 | 11245 | 1335 | 74153 | 2312 |
| **Maternal educational level** |  |  |  |  |  |  |  |  |
| Low (n, %) | 1751 (16.9%) | 1363 (56.3%) | 130 (18.2%) | 7369  (13.4%) | 499 (4.4%) | 272 (20.4%) | 1497 (2.0%) | 1208 (52.2%) |
| Medium (n, %) | 7122 (68.9%) | 336 (13.9%) | 362 (50.8%) | 20315 (36.8%) | 3120 (27.7%) | 547 (41.0%) | 22629 (30.5%) | 631 (27.3%) |
| High (n, %) | 1466 (14.2%) | 722 (29.8%) | 221 (31.0%) | 27476 (49.8%) | 7626 (67.8%) | 516 (38.7%) | 50027 (67.5%) | 473 (20.5%) |
| **Externalising problems** |  |  |  |  |  |  |  |  |
| Scores < the 90^th^ percentile^*^ (n, %) | 9288 (89.8%) | 2112 (87.2%) | 646 (90.6%) | 49607 (89.9%) | 9940 (88.4%) | 1197 (89.7%) | 66129 (89.2%) | 2075 (89.7%) |
| Scores ≥ the 90^th^ percentile^*^ (n, %) | 1051 (10.2%) | 309  (12.8%) | 67  (9.4%) | 5553 (10.1%) | 1305 (11.6%) | 138  (10.3%) | 8024 (10.8%) | 237  (10.3%) |
| **Internalising problems** |  |  |  |  |  |  |  |  |
| Scores < the 90^th^ percentile^*^ (n, %) | 9191 (88.9%) | 2131 (88.0%) | 642 (90.0%) | 49288 (89.4%) | 9930 (88.3%) | 1199 (89.8%) | 64476 (86.9%) | 2045 (88.5%) |
| Scores ≥ the 90^th^ percentile^*^ (n, %) | 1148 (11.1%) | 290 (12.0%) | 71 (10.0%) | 5872 (10.6%) | 1315 (11.7%) | 136 (10.2%) | 9677 (13.1%) | 267  (11.5%) |
| **Sex,** Male (n, %) |  |  |  |  |  |  |  |  |
| Imputed dataset 1 | 5324 (51.5%) | 1212 (50.1%) | 333  (46.7%) | 27314 (49.5%) | 5738 (51.0%) | 667 (50.0%) | 37957 (51.2%) | 1187 (51.3%) |
| Imputed dataset 2 | 5324 (51.5%) | 1212 (50.1%) | 333  (46.7%) | 27314 (49.5%) | 5737 (51.0%) | 667 (50.0%) | 37957 (51.2%) | 1187 (51.3%) |
| Imputed dataset 3 | 5324 (51.5%) | 1212 (50.1%) | 333  (46.7%) | 27314 (49.5%) | 5736 (51.0%) | 667 (50.0%) | 37957 (51.2%) | 1187 (51.3%) |
| Imputed dataset 4 | 5324 (51.5%) | 1212 (50.1%) | 333  (46.7%) | 27314 (49.5%) | 5739 (51.0%) | 667 (50.0%) | 37957 (51.2%) | 1187 (51.3%) |
| Imputed dataset 5 | 5324 (51.5%) | 1212 (50.1%) | 333  (46.7%) | 27314 (49.5%) | 5736 (51.0%) | 667 (50.0%) | 37957 (51.2%) | 1187 (51.3%) |
| **Maternal age at birth, in years,** Mean (SD) |  |  |  |  |  |  |  |  |
| Imputed dataset 1 | 28.8 (4.6) | 28.5 (5.5) | 30.8 (4.6) | 30.2 (4.2) | 30.9 (4.7) | 32.1 (3.9) | 30.4 (4.4) | 27.9 (5.8) |
| Imputed dataset 2 | 28.7 (4.6) | 28.5 (5.5) | 30.8 (4.6) | 30.2 (4.2) | 30.9 (4.7) | 32.1 (3.9) | 30.4 (4.4) | 27.9 (5.8) |
| Imputed dataset 3 | 28.8 (4.6) | 28.5 (5.5) | 30.8 (4.6) | 30.2 (4.2) | 30.9 (4.7) | 32.1 (3.9) | 30.4 (4.4) | 27.9 (5.8) |
| Imputed dataset 4 | 28.7 (4.6) | 28.5 (5.5) | 30.8 (4.6) | 30.2 (4.2) | 30.9 (4.7) | 32.1 (3.9) | 30.4 (4.4) | 27.9 (5.8) |
| Imputed dataset 5 | 28.8 (4.6) | 28.5 (5.5) | 30.8 (4.6) | 30.2 (4.2) | 30.9 (4.7) | 32.1 (3.9) | 30.4 (4.4) | 27.9 (5.8) |
| **Maternal smoking in pregnancy,** Yes (n, %) |  |  |  |  |  |  |  |  |
| Imputed dataset 1 | 2589 (25.0%) | 230 (9.5%) | 201 (28.2%) | 13453 (24.4%) | 1865 (16.6%) | 377 (28.2%) | 15793 (21.3%) | 639 (27.6%) |
| Imputed dataset 2 | 2573 (24.9%) | 230 (9.5%) | 202 (28.3%) | 13456 (24.4%) | 1858 (16.5%) | 379 (28.4%) | 15793 (21.3%) | 639 (27.6%) |
| Imputed dataset 3 | 2601 (25.2%) | 231 (9.5%) | 201 (28.2%) | 13452 (24.4%) | 1861 (16.5%) | 378 (28.3%) | 15793 (21.3%) | 639 (27.6%) |
| Imputed dataset 4 | 2599 (25.1%) | 231 (9.5%) | 201 (28.2%) | 13457 (24.4%) | 1869 (16.6%) | 381 (28.5%) | 15793 (21.3%) | 639 (27.6%) |
| Imputed dataset 5 | 2581 (25.0%) | 230 (9.5%) | 201 (28.2%) | 13473 (24.4%) | 1864 (16.6%) | 383 (28.7%) | 15793 (21.3%) | 639 (27.6%) |
| **Gestational age, in days,** Mean (SD) |  |  |  |  |  |  |  |  |
| Imputed dataset 1 | 279.5 (12.6) | 276.1 (13.0) | 282.9  (8.5) | 279.9 (11.9) | 277.5  (9.9) | 279.3 (10.4) | 278.8 (13.1) | 273.8 (16.1) |
| Imputed dataset 2 | 279.5 (12.6) | 276.1 (12.9) | 282.8  (8.5) | 279.9 (11.9) | 277.5  (9.9) | 279.4 (10.4) | 278.8 (13.1) | 273.8 (16.1) |
| Imputed dataset 3 | 279.5 (12.6) | 276.1 (13.0) | 282.9  (8.5) | 279.9 (11.9) | 277.5  (9.9) | 279.3 (10.5) | 278.8 (13.1) | 273.8 (16.1) |
| Imputed dataset 4 | 279.5 (12.6) | 276.1 (13.0) | 282.8  (8.5) | 279.9 (11.9) | 277.5  (9.9) | 279.4 (10.4) | 278.8 (13.1) | 273.8 (16.1) |
| Imputed dataset 5 | 279.5 (12.6) | 276.1 (12.9) | 282.8  (8.5) | 279.9 (11.9) | 277.5  (9.9) | 279.3 (10.4) | 278.8 (13.1) | 273.9 (16.1) |
| **Small for gestational age,** Yes (n, %); missing (n, %) |  |  |  |  |  |  |  |  |
| Imputed dataset 1 | 794 (7.7%);  35  (0.3%) | 349 (14.4%);  2  (0.1%) | 66 (9.3%);  1  (0.1%) | 1886 (3.4%); 214 (0.4%) | 757 (6.7%);  2  (0.0%) | 137 (10.3%);  3  (0.2%) | 2259 (3.0%); 132  (0.2%) | 126 (5.4%);  4  (0.2%) |
| Imputed dataset 2 | 793 (7.7%);  35  (0.3%) | 347 (14.3%);  2  (0.1%) | 66 (9.3%);  1  (0.1%) | 1884 (3.4%); 214 (0.4%) | 760 (6.8%);  1  (0.0%) | 138 (10.3%);  3  (0.2%) | 2252 (3.0%); 132  (0.2%) | 129 (5.6%);  4  (0.2%) |
| Imputed dataset 3 | 792 (7.7%);  35  (0.3%) | 345 (14.3%);  2  (0.1%) | 67 (9.4%);  1  (0.1%) | 1884 (3.4%); 214 (0.4%) | 755 (6.7%);  1  (0.0%) | 138 (10.3%);  3  (0.2%) | 2265 (3.1%); 133  (0.2%) | 132 (5.7%);  4  (0.2%) |
| Imputed dataset 4 | 794 (7.7%);  35  (0.3%) | 345 (14.3%);  2  (0.1%) | 66 (9.3%);  1  (0.1%) | 1885 (3.4%); 214 (0.4%) | 760 (6.8%);  1  (0.0%) | 137 (10.3%);  3  (0.2%) | 2284 (3.1%); 134  (0.2%) | 130 (5.6%);  4  (0.2%) |
| Imputed dataset 5 | 793 (7.7%);  35  (0.3%) | 348 (14.4%);  2  (0.1%) | 66 (9.3%);  1  (0.1%) | 1886 (3.4%); 214 (0.4%) | 759 (6.7%);  1  (0.0%) | 137 (10.3%);  3  (0.2%) | 2244 (3.0%); 134  (0.2%) | 130 (5.6%);  4  (0.2%) |
| **Postpartum depression,** Yes (n, %) |  |  |  |  |  |  |  |  |
| Imputed dataset 1 | 852 (8.2%) | 587  (24.2%) | 93  (13.0%) | 8559 (15.5%) | 985 (8.8%) | 157  (11.8%) | 7446 (10.0%) | 272  (11.8%) |
| Imputed dataset 2 | 875 (8.5%) | 652  (26.9%) | 82  (11.5%) | 8427 (15.3%) | 973 (8.7%) | 157  (11.8%) | 7476 (10.1%) | 239 (10.3%) |
| Imputed dataset 3 | 865 (8.4%) | 548  (22.6%) | 85  (11.9%) | 8521 (15.4%) | 974 (8.7%) | 166  (12.4%) | 7454 (10.1%) | 231 (10.0%) |
| Imputed dataset 4 | 864 (8.4%) | 590  (24.4%) | 90  (12.6%) | 8442 (15.3%) | 986 (8.8%) | 144  (10.8%) | 7465 (10.1%) | 238 (10.3%) |
| Imputed dataset 5 | 872 (8.4%) | 637  (26.3%) | 90 (12.6%) | 8486 (15.4%) | 979 (8.7%) | 185  (13.9%) | 7458 (10.1%) | 247 (10.7%) |
| **Child ever breast fed,** Yes (n, %) |  |  |  |  |  |  |  |  |
| Imputed dataset 1 | 7805 (75.5%) | 1968 (81.3%) | 523 (73.4%) | 54216 (98.3%) | 8522 (75.8%) | 1198 (89.7%) | 69355 (93.5%) | 2080 (90.0%) |
| Imputed dataset 2 | 7784 (75.3%) | 1968 (81.3%) | 523 (73.4%) | 54194 (98.2%) | 8505 (75.6%) | 1200 (89.9%) | 69342 (93.5%) | 2075 (89.7%) |
| Imputed dataset 3 | 7811 (75.5%) | 1968 (81.3%) | 523 (73.4%) | 54191 (98.2%) | 8504 (75.6%) | 1198 (89.7%) | 69352 (93.5%) | 2081 (90.0%) |
| Imputed dataset 4 | 7812 (75.6%) | 1968 (81.3%) | 523 (73.4%) | 54189 (98.2%) | 8501 (75.6%) | 1196 (89.6%) | 69359 (93.5%) | 2078 (89.9%) |
| Imputed dataset 5 | 7818 (75.6%) | 1968 (81.3%) | 523 (73.4%) | 54190 (98.2%) | 8518 (75.7%) | 1197  (89.7%) | 69364 (93.5%) | 2077 (89.8%) |

^*^ Using the 90^th^ percentile values of the sample with complete cases on the exposure and the outcome variables of each cohort

**Table S4:** Slope index of inequality and 95% CI between scaled maternal education and each mediator and confounder and between scaled maternal education and externalising and internalising problems for each imputed dataset. Pooled SIIs are estimated from the 5 SIIs of the imputed datasets within each cohort using the Rubin’s Rule. Combined SIIs are estimated as the weighted average of the SIIs of each imputed dataset across all cohorts.

|  | **ALSPAC** | **BiB** | **CHOP** | **DNBC** | **ELFE** | **INMA** | **MoBa** | **Raine Study** | **Combined** |
| --- | --- | --- | --- | --- | --- | --- | --- | --- | --- |
| **Maternal smoking in pregnancy** |  |  |  |  |  |  |  |  |  |
| Imputed dataset 1 | -43.3  [-52.8, -33.8] | -12.9  [-39.6, 13.9] | -47.5  [-56.3, -38.8] | -30.4  [-34.3, -26.5] | -31.5  [-34.6, -28.3] | -33.2  [-37.2, -29.3] | -52.5  [-79.1, -25.8] | -29.3  [-49.6, -9.0] | -41.5 [-54.2, -28.9] |
| Imputed dataset 2 | -42.2  [-51.9, -32.6] | -13.1  [-39.7, 13.6] | -47.0 [-56.3, -37.7] | -30.3  [-33.6, -26.9] | -30.9  [-33.4, -28.5] | -33.1  [-38.5, -27.7] | -52.6  [-79.5, -25.7] | -29.1  [-49.3, -8.9] | -41.4 [-54.1, -28.7] |
| Imputed dataset 3 | -42.1  [-51.1, -33.1] | -13.0  [-40.8, 14.8] | -47.7 [-56.3, -39.1] | -30.3  [-33.6, -26.9] | -30.8  [-32.4, -29.1] | -33.9  [-38.3, -29.6] | -52.6  [-79.5, -25.7] | -29.1  [-49.3, -8.9] | -41.4 [-54.1, -28.7] |
| Imputed dataset 4 | -42.3  [-51.6, -33.0] | -13.0  [-40.8, 14.8] | -47.7 [-56.3, -39.1] | -30.4  [-34.0, -26.9] | -31.5  [-34.3, -28.7] | -33.8  [-37.0, -30.5] | -52.6  [-79.5, -25.7] | -29.1  [-49.3, -8.9] | -41.5 [-54.2, -28.8] |
| Imputed dataset 5 | -42.6  [-51.3, -34.0] | -13.1  [-39.7, 13.6] | -47.7 [-56.3, -39.1] | -30.3  [-33.9, -26.6] | -31.0  [-33.2, -28.8] | -34.0  [-36.2, -31.8] | -52.6  [-79.5, -25.7] | -29.1  [-49.3, -8.9] | -41.4 [-54.2, -28.7] |
| Pooled | -42.5  [-51.8, -33.2] | -13.0  [-40.1, 14.2] | -47.5  [-56.3, -38.7] | -30.3  [-33.9, -26.8] | -31.1  [-33.8, -28.5] | -33.6  [-37.7, -29.5] | -52.5  [-79.4, -25.7] | -29.2  [-49.4, -8.9] |  |
| **Gestational age** |  |  |  |  |  |  |  |  |  |
| Imputed dataset 1 | 0.5  [-0.8, 1.7] | -0.1  [-1.3, 1.1] | -1.8  [-5.1, 1.4] | 1.0  [0.5, 1.4] | 2.4  [1.2, 3.5] | -0.1  [-2.4, 2.1] | 2.9  [0.5, 5.2] | 1.4  [-0.5, 3.4] | 1.9  [0.8, 3.0] |
| Imputed dataset 2 | 0.3  [-1.1, 1.7] | 0.0  [-1.1, 1.2] | -1.7  [-5.2, 1.8] | 0.9  [0.5, 1.4] | 2.3  [1.2, 3.4] | -0.3  [-2.5, 2.0] | 2.8  [0.5, 5.1] | 2.3  [-0.7, 5.2] | 1.9  [0.8, 3.0] |
| Imputed dataset 3 | 0.4  [-1.0, 1.7] | 0.1  [-1.5, 1.6] | -1.7  [-5.0, 1.7] | 0.9  [0.5, 1.4] | 2.4  [1.1, 3.7] | -0.1  [-2.5, 2.2] | 2.8  [0.6, 5.0] | 2.1  [-0.8, 4.9] | 1.9  [0.8, 2.9] |
| Imputed dataset 4 | 0.4  [-1.0, 1.8] | 0.1  [-1.4, 1.7] | -1.7  [-5.1, 1.7] | 0.9  [0.5, 1.4] | 2.5  [1.2, 3.8] | -0.2  [-2.4, 2.0] | 2.7  [0.7, 4.8] | 2.1  [-1.0, 5.1] | 1.8  [0.9, 2.8] |
| Imputed dataset 5 | 0.4  [-1.0, 1.8] | 0.1  [-1.0, 1.2] | -1.7  [-5.2, 1.8] | 0.9  [0.5, 1.4] | 2.4  [1.4, 3.3] | -0.1  [-2.3, 2.0] | 2.9  [0.5, 5.4] | 2.1  [-0.8, 5.0] | 1.9  [0.7, 3.1] |
| Pooled | 0.4  [-1.0, 1.8] | 0.0  [-1.3, 1.4] | -1.7  [-5.1, 1.7] | 1.0  [0.5, 1.4] | 2.4  [1.2, 3.6] | -0.2  [-2.4, 2.1] | 2.8  [0.5, 5.1] | 2.0  [-0.9, 4.8] |  |
| **Small for gestational age** |  |  |  |  |  |  |  |  |  |
| Imputed dataset 1 | -4.8  [-5.3, -4.4] | -0.6  [-6.6, 5.4] | -13.4  [-29.8, 2.9] | -2.0  [-2.2, -1.7] | -4.5  [-5.4, -3.6] | -6.3  [-9.6, -3.1] | -1.4  [-3.9, 1.2] | -3.5  [-5.0, -2.0] | -2.1  [-3.4, -0.9] |
| Imputed dataset 2 | -4.9  [-5.0, -4.8] | -0.2  [-5.9, 5.5] | -13.4  [-29.8, 2.9] | -2.0  [-2.2, -1.7] | -4.6  [-6.3, -2.9] | -6.1  [-9.6, -2.6] | -0.9  [-2.5, 0.7] | -3.9  [-5.8, -2.1] | -1.9  [-2.7, -1.2] |
| Imputed dataset 3 | -5.2  [-5.2, -5.1] | -0.7  [-5.7, 4.3] | -13.4  [-29.0, 2.1] | -2.0  [-2.3, -1.7] | -4.5  [-5.5, -3.6] | -6.4  [-9.3, -3.4] | -1.1  [-3.2, 1.1] | -4.6  [-5.8, -3.4] | -2.0  [-3.1, -1.0] |
| Imputed dataset 4 | -5.1  [-5.1, -5.0] | -0.6  [-4.4, 3.2] | -13.4  [-29.8, 2.9] | -1.9  [-2.2, -1.6] | -4.4  [-5.5, -3.3] | -6.3  [-9.6, -3.1] | -1.5  [-4.2, 1.1] | -3.5  [-5.1, -1.8] | -2.2  [-3.5, -1.0] |
| Imputed dataset 5 | -5.1  [-5.2, -5.0] | -0.4  [-6.3, 5.5] | -13.4  [-29.8, 2.9] | -2.0  [-2.4, -1.6] | -4.5  [-5.3, -3.6] | -6.3  [-9.6, -3.1] | -1.0  [-2.8, 0.8] | -4.5  [-6.1, -2.9] | -2.0  [-2.9, -1.1] |
| Pooled | -5.0  [-5.4, -4.7] | -0.5  [-5.9, 4.8] | -13.4  [-29.6, 2.8] | -2.0  [-2.3, -1.6] | -4.5  [-5.7, -3.3] | -6.3  [-9.5, -3.0] | -1.2  [-3.4, 1.1] | -4.0  [-5.9, -2.0] |  |
| **Postpartum depression** |  |  |  |  |  |  |  |  |  |
| Imputed dataset 1 | -5.5  [-11.7, 0.6] | -1.2  [-34.7, 32.2] | -2.0  [-2.6, -1.4] | -5.4  [-9.5, -1.3] | -11.4  [-25.7, 2.9] | -11.7  [-13.0, -10.5] | -17.4  [-33.1, -1.8] | -3.5  [-12.9, 5.9] | -11.4  [-19.0, -3.8] |
| Imputed dataset 2 | -6.5  [-13.7, 0.6] | -8.3  [-65.5, 48.9] | -1.9  [-2.0, -1.7] | -5.6  [-9.2, -1.9] | -10.8  [-23.6, 2.0] | -13.6  [-16.8, -10.4] | -18.8  [-37.3, -0.2] | -6.9  [-7.1, -6.7] | -12.3  [-21.3, -3.4] |
| Imputed dataset 3 | -5.9  [-12.5, 0.8] | 1.7  [-19.0, 22.5] | -1.9  [-4.1, 0.3] | -5.3  [-8.3, -2.4] | -10.3  [-22.1, 1.5] | -5.4  [-9.6, -1.2] | -17.9  [-34.5, -1.3] | -1.2  [-11.5, 9.0] | -11.5  [-19.4, -3.5] |
| Imputed dataset 4 | -5.6  [-12.3, 1.2] | 0.5  [-6.5, 7.5] | -2.1  [-4.1, -0.1] | -4.4  [-7.7, -1.1] | -12.8  [-29.1, 3.6] | -10.1  [-12.3, -7.9] | -18.0  [-34.9, -1.1] | -3.7  [-12.0, 4.6] | -11.4  [-19.5, -3.3] |
| Imputed dataset 5 | -6.2  [-13.2, 0.7] | 6.5  [-6.8, 19.7] | -0.2  [-2.4, 2.0] | -5.2  [-9.4, -1.0] | -10.6  [-22.5, 1.4] | -20.4  [-23.3, -17.5] | -18.8  [-37.6, 0.1] | -3.4  [-5.0, 1.8] | -11.9  [-21.0, -2.9] |
| Pooled | -6.0  [-12.8, 0.8] | -0.2  [-34.0, 33.6] | -1.6  [-4.0, 0.8] | -5.2  [-9.0, -1.4] | -11.2  [-24.9, 2.5] | -12.2  [-24.3, -0.1] | -18.2  [-35.6, -0.8] | -3.8  [-12.3, 4.7] |  |
| **Child ever breast fed** |  |  |  |  |  |  |  |  |  |
| Imputed dataset 1 | 46.7  [45.1, 48.2] | 31.3  [23.0, 39.6] | 34.6  [21.8, 47.3] | 2.6  [2.1, 3.0] | 19.8  [10.7, 28.9] | 10.5  [9.8, 11.2] | 6.3  [2.6, 10.1] | 10.5  [-3.6, 24.6] | 9.2  [7.3, 11.2] |
| Imputed dataset 2 | 46.7  [45.1, 48.2] | 31.1  [22.6, 39.6] | 34.7  [22.1, 47.3] | 2.6  [2.5, 2.7] | 21.3  [17.0, 25.6] | 11.0  [9.8, 12.1] | 7.0  [1.8, 12.3] | 11.4  [-2.5, 25.2] | 9.7  [7.2, 12.2] |
| Imputed dataset 3 | 47.1  [44.0, 50.3] | 31.1  [22.6, 39.6] | 34.7  [22.1, 47.3] | 2.6  [2.5, 2.7] | 20.1  [13.0, 27.3] | 11.5  [10.5, 12.6] | 5.6  [3.3, 8.0] | 9.6  [-3.5, 22.7] | 9.0  [7.7, 10.2] |
| Imputed dataset 4 | 46.2  [45.2, 47.2] | 31.1  [22.6, 39.6] | 34.7  [22.1, 47.3] | 2.9  [2.3, 3.4] | 20.7  [12.7, 28.8] | 11.3  [9.9, 12.7] | 6.5  [2.4, 10.5] | 10.6  [-4.9, 26.1] | 9.4  [7.4, 11.4] |
| Imputed dataset 5 | 47.0  [45.1, 49.0] | 31.1  [22.6, 39.6] | 34.7  [22.1, 47.3] | 2.9  [2.3, 3.4] | 20.0  [12.9, 27.0] | 10.9  [10.8, 11.1] | 7.2  [1.2, 13.2] | 10.1  [-5.4, 25.6] | 9.8  [6.9, 12.7] |
| Pooled | 46.7  [44.6, 48.9] | 31.2  [22.7, 39.6] | 34.7  [22.0, 47.3] | 2.7  [2.2, 3.2] | 20.4  [13.0, 27.8] | 11.0  [9.8, 12.3] | 6.5  [1.9, 11.2] | 10.4  [-4.1, 24.9] |  |
| **Maternal age at birth** |  |  |  |  |  |  |  |  |  |
| Imputed dataset 1 | 4.6  [1.8, 7.4] | 3.4  [-1.2, 8.0] | 2.3  [1.2, 3.4] | 4.1  [2.5, 5.7] | 2.7  [-0.4, 5.8] | 1.6  [1.1, 2.1] | 5.3  [3.9, 6.6] | 6.0  [1.5, 10.4] | 4.6  [3.7, 5.5] |
| Imputed dataset 2 | 4.6  [1.7, 7.6] | 3.4  [-1.2, 8.0] | 2.3  [1.2, 3.4] | 4.1  [2.5, 5.7] | 2.7  [-0.4, 5.8] | 1.7  [1.1, 2.2] | 5.2  [4.0, 6.5] | 6.0  [1.6, 10.3] | 4.6  [3.7, 5.4] |
| Imputed dataset 3 | 4.6  [1.6, 7.6] | 3.4  [-1.2, 8.0] | 2.3  [1.2, 3.4] | 4.1  [2.5, 5.7] | 2.7  [-0.5, 5.8] | 1.6  [1.0, 2.2] | 5.2  [4.0, 6.5] | 6.0  [1.6, 10.3] | 4.6  [3.7, 5.4] |
| Imputed dataset 4 | 4.6  [1.8, 7.5] | 3.4  [-1.2, 8.0] | 2.3  [1.2, 3.4] | 4.1  [2.5, 5.7] | 2.7  [-0.4, 5.8] | 1.6  [1.1, 2.1] | 5.3  [3.9, 6.6] | 6.0  [1.7, 10.3] | 4.6  [3.7, 5.5] |
| Imputed dataset 5 | 4.6  [1.6, 7.6] | 3.4  [-1.2, 8.0] | 2.3  [1.2, 3.4] | 4.1  [2.5, 5.7] | 2.7  [-0.4, 5.8] | 1.6  [1.1, 2.2] | 5.3  [3.9, 6.7] | 5.9  [1.6, 10.2] | 4.6  [3.7, 5.5] |
| Pooled | 4.6  [1.7, 7.5] | 3.4  [-1.2, 8.0] | 2.3  [1.2, 3.4] | 4.1  [2.5, 5.7] | 2.7  [-0.4, 5.8] | 1.6  [1.1, 2.2] | 5.3  [3.9, 6.6] | 6.0  [1.6, 10.3] |  |
| **Externalising problems** |  |  |  |  |  |  |  |  |  |
| Imputed dataset 1 | -9.0  [-11.4, -6.7] | -15.6  [-25.5, -5.7] | -20.8  [-24.1, -17.6] | -10.6  [-12.8, -8.3] | -9.7  [-16.6, -2.8] | -11.3  [-16.6, -6.0] | -14.4  [-25.2, -3.5] | -7.8  [-11.8, -3.8] | -12.3  [-17.5, -7.1] |
| Imputed dataset 2 | -9.3  [-11.4, -7.2] | -15.6  [-25.5, -5.8] | -20.9  [-24.1, -17.7] | -10.5  [-12.8, -8.3] | -9.7  [-16.6, -2.8] | -10.9  [-16.0, -5.9] | -14.4  [-25.3, -3.5] | -7.7  [-11.6, -3.7] | -12.3  [-17.5, -7.1] |
| Imputed dataset 3 | -9.3  [-11.4, -7.2] | -15.6  [-25.5, -5.8] | -20.9  [-24.1, -17.7] | -10.5  [-12.8, -8.3] | -9.7  [-16.6, -2.8] | -10.9  [-16.0, -5.9] | -14.4  [-25.3, -3.5] | -7.7  [-11.6, -3.7] | -12.3  [-17.5, -7.1] |
| Imputed dataset 4 | -9.3  [-11.4, -7.2] | -15.6  [-25.5, -5.8] | -20.9  [-24.1, -17.7] | -10.5  [-12.8, -8.3] | -9.7  [-16.6, -2.8] | -10.9  [-16.0, -5.9] | -14.4  [-25.3, -3.5] | -7.7  [-11.6, -3.7] | -12.3  [-17.5, -7.1] |
| Imputed dataset 5 | -9.3  [-11.4, -7.2] | -15.6  [-25.5, -5.8] | -20.9  [-24.1, -17.7] | -10.5  [-12.8, -8.3] | -9.7  [-16.6, -2.8] | -10.9  [-16.0, -5.9] | -14.4  [-25.3, -3.5] | -7.7  [-11.6, -3.7] | -12.3  [-17.5, -7.1] |
| Pooled | -9.2  [-11.4, -7.1] | -15.6  [-25.5, -5.7] | -20.9  [-24.1, -17.6] | -10.5  [-12.8, -8.3] | -9.7  [-16.6, -2.8] | -11.0  [-16.1, -5.9] | -14.4  [-25.3, -3.5] | -7.7  [-11.6, -3.7] |  |
| **Internalising problems** |  |  |  |  |  |  |  |  |  |
| Imputed dataset 1 | -5.1  [-7.3, -2.9] | -8.8  [-17.8, 0.1] | -15.1  [-19.5, -10.7] | -8.7  [-9.2, -8.1] | -13.1  [-17.3, -8.9] | -12.8  [-20.1, -5.4] | -13.9  [-28.9, 1.1] | -2.9  [-4.6, -1.3] | -11.2  [-18.2, -4.1] |
| Imputed dataset 2 | -5.1  [-7.3, -3.0] | -8.8  [-17.8, 0.1] | -15.1  [-19.5, -10.8] | -8.7  [-9.2, -8.1] | -13.1  [-17.3, -8.9] | -12.9  [-21.1, -4.7] | -14.0  [-28.9, 0.9] | -2.8  [-4.3, -1.3] | -11.3  [-18.3, -4.2] |
| Imputed dataset 3 | -5.1  [-7.3, -3.0] | -8.8  [-17.8, 0.1] | -15.1  [-19.5, -10.8] | -8.7  [-9.2, -8.1] | -13.1  [-17.3, -8.9] | -12.9  [-21.1, -4.7] | -14.0  [-28.9, 0.9] | -2.8  [-4.3, -1.3] | -11.3  [-18.3, -4.2] |
| Imputed dataset 4 | -5.1  [-7.3, -3.0] | -8.8  [-17.8, 0.1] | -15.1  [-19.5, -10.8] | -8.7  [-9.2, -8.1] | -13.1  [-17.3, -8.9] | -12.9  [-21.1, -4.7] | -14.0  [-28.9, 0.9] | -2.8  [-4.3, -1.3] | -11.3  [-18.3, -4.2] |
| Imputed dataset 5 | -5.1  [-7.3, -3.0] | -8.8  [-17.8, 0.1] | -15.1  [-19.5, -10.8] | -8.7  [-9.2, -8.1] | -13.1  [-17.3, -8.9] | -12.9  [-21.1, -4.7] | -14.0  [-28.9, 0.9] | -2.8  [-4.3, -1.3] | -11.3  [-18.3, -4.2] |
| Pooled | -5.1  [-7.3, -3.0] | -8.8  [-17.8, 0.1] | -15.1  [-19.5, -10.8] | -8.7  [-9.2, -8.1] | -13.1  [-17.3, -8.9] | -12.9  [-20.9, -4.9] | -14.0  [-28.9, 0.9] | -2.8  [-4.4, -1.3] |  |

The pooled slope index of inequality for the $m=5$ imputed datasets is given as the mean of the 5 SIIs, where the pooled standard error (SE) of SII is given as the square root of the within variance plus the between variance times $(m+1)/m$:

$$SII_{pooled}=\frac{1}{m}\sum_{i=1}^{m} SII_{i}$$

$$SE\left( SII_{pooled} \right)=\sqrt{within variance+\left( \frac{m+1}{m} \right)*between variance}$$

$$within variance=\frac{1}{m}\sum_{i=1}^{m} \left( SE\left( SII_{i} \right) \right)^{2}$$

$$between variance=\sum_{i=1}^{m} \left( SII_{i}-SII_{pooled} \right)^{2}$$

$$95\%CI of SII_{pooled}= SII_{pooled}\pm1.96*SE\left( SII_{pooled} \right)$$

***Robustness tests: Selection of the age when the outcome is measured:***

Most of the participating cohorts collected data for externalising and internalising problems at multiple data collection waves (i.e. at different ages of the child; see Figure S1). For our main analysis we used one score per child that was closer to age 5 which was selected from the wider age interval of 0-20 years. While most of the children had a score close to age 5, 10339 children across all cohorts (7.7% of the combined total sample) had available scores only at age 11 and above. In general, this should not be problematic given that the used percentile scores for the outcome variables have been calculated by cohorts separately at each age (giving a percentage score for each child compared to their peers of the same age). However, we excluded the 7.7% of children with age of the outcome measure greater or equal to 11 and recalculated the inequalities using the rest 93.3% of the sample as a sensitivity analysis.

The exclusion of the children that had the outcome scores measured only at ages above age 10, slightly reduced the complete cases sample with the biggest reduction observed in the DNBC cohort (around 22%), (see Figure S10 and Table S5). This exclusion did not change the median and the interquartile range of child age when the outcome is measured (see Table S6). This sensitivity analysis also gave similar SIIs compared to the main analysis (compare Figures S11 and S12 with Figures 3 and S2 and Figure S13 with Figure 4). The weighted average of SIIs across all cohorts was -12.9 and -11.2 for externalising and internalising problems respectively (Figure S13) in the sensitivity analysis while the corresponding values in the main analysis were -12.0 and -10.9 respectively (Figure 4).

**Figure S10:** Frequency of externalising (red colour) and internalising (green colour) scores at the child ages when the outcome variables are selected for the main analysis. In a sensitivity analysis we excluded the children with externalising/internalising scores measured above age 10.

**Table S5:** Complete cases sample size

|  | **ALSPAC** | **BiB** | **CHOP** | **DNBC** | **ELFE** | **INMA** | **MoBa** | **Raine Study** |
| --- | --- | --- | --- | --- | --- | --- | --- | --- |
| In the main analysis | 8144 | 450 | 639 | 45190 | 10397 | 798 | 67389 | 1400 |
| In the sensitivity analysis | 8061 | 450 | 611 | 35066 | 10397 | 740 | 67389 | 1381 |

**Table S6:** Child age in years (median and interquartile range (IQR)) when the outcome variables are measured

|  | **ALSPAC** | **BiB** | **CHOP** | **DNBC** | **ELFE** | **INMA** | **MoBa** | **Raine**  **Study** |
| --- | --- | --- | --- | --- | --- | --- | --- | --- |
| **Child age, median [IQR]** |  |  |  |  |  |  |  |  |
| In the main analysis | 4 [4-6] | 4 [4-4] | 5 [5-5] | 7 [7-7] | 5 [5-6] | 8 [7-9] | 5 [3-5] | 5 [5-5] |
| In the sensitivity analysis | 4 [4-6] | 4 [4-4] | 5 [5-5] | 7 [7-7] | 5 [5-6] | 8 [7-9] | 5 [3-5] | 5 [5-5] |

**Figure S11:** Prevalence or mean of early-life factors at each level of scaled maternal education using their complete cases derived from the narrowed age interval used for the selection of the outcome variable. The slope of each trendline defines the Slope Index of Inequality.

**Figure S12:** Slope Index of Inequalities between scaled maternal education and early-life risk factors as calculated based on trendlines in Figure S11. The combined SII in each panel is calculated as the weighted average of the cohort-specific SIIs. The weights are defined by each pairwise complete cases sample size of each cohort.

**Figure S13:** Prevalence of externalising (A) and internalising problems (B) at each level of scaled maternal education using the maternal education and the outcome variables scaled and dichotomised respectively using the sample sizes of the complete cases derived from the narrowed age interval used for the selection of the outcome variable. The slope of each trendline defines the Slope Index of Inequality. The forest plots summarise the SIIs and their 95% confidence intervals for externalising (C) and internalising (D) problems. The combined SII is calculated as the weighted average of the cohort-specific SIIs where the weights are defined by each pairwise complete cases sample size of each cohort.

***Robustness tests: Child’s sex as an effect modifier***

Child’s sex is not a confounder in the examined relationship since it does not affect maternal education. However, we explored if child’s sex is an effect modifier for the relationship between maternal education and externalising and internalising problems. In the regression models, we included an interaction term between scaled maternal education and child’s sex to test its significance using Wald Test p-values. We were not able to apply Likelihood Ratio Tests to compare the goodness of fit between the model with the interaction term and the model with no interaction due to unavailability of such functionality in DataSHIELD.

We found no evidence that child’s sex is an effect modifier (all p-values of the interaction term > 0.05) of the relationship between maternal education and children’s externalising and internalising problems (Table S7). Furthermore, we calculated social inequalities in mediators and outcomes in stratified datasets (one for males and one for females) and we did not observe any significant differences either between them or compared with the results of the main analysis (see Figures S14-S19).

**Table S7:** Wald test p-values of the estimates of the interaction term examining whether child’s sex is an effect modifier for the relationship between maternal education and externalising or internalising problems.

|  | **ALSPAC** | **BiB** | **CHOP** | **DNBC** | **ELFE** | **INMA** | **MoBa** | **Raine Study** |
| --- | --- | --- | --- | --- | --- | --- | --- | --- |
| **Externalising problems** |  |  |  |  |  |  |  |  |
| Model adjusted by confounder | 0.20 | 0.45 | 0.44 | 0.75 | 0.62 | 0.74 | 0.34 | 0.36 |
| Model adjusted by mediators and confounder | 0.21 | 0.43 | 0.43 | 0.73 | 0.55 | 0.95 | 0.33 | 0.49 |
| **Internalising problems** |  |  |  |  |  |  |  |  |
| Model adjusted by confounder | 0.19 | 0.91 | 0.99 | 0.28 | 0.21 | 0.70 | 0.83 | 0.32 |
| Model adjusted by mediators and confounder | 0.21 | 0.98 | 1 | 0.29 | 0.26 | 0.65 | 0.76 | 0.45 |

**Figure S14:** Prevalence or mean of early-life factors at each level of scaled maternal education using the complete cases samples for **male** children. The slope of each trendline defines the Slope Index of Inequality. To avoid any disclosure of individual participant data, BiB cohort is not presented in the panels of maternal smoking in pregnancy and child ever breast fed because there are less than 3 children with positive or negative answers in one maternal educational level for these two variables.

**Figure S15:** Slope Index of Inequalities between scaled maternal education and early-life risk factors as calculated based on trendlines in Figure S14. The combined SII in each panel is calculated as the weighted average of the cohort-specific SIIs. The weights are defined by each pairwise complete cases sample size of each cohort. To avoid any disclosure of individual participant data, BiB cohort is not presented in the panels of maternal smoking in pregnancy and child ever breast fed because there are less than 3 children with positive or negative answers in one maternal educational level for these two variables.

**Figure S16:** Prevalence of externalising (A) and internalising problems (B) at each level of scaled maternal education using the maternal education and the outcome variables scaled and dichotomised respectively using the sample sizes of the complete cases samples for **male** children. The slope of each trendline defines the Slope Index of Inequality. The forest plots summarise the SIIs and their 95% confidence intervals for externalising (C) and internalising (D) problems. The combined SII is calculated as the weighted average of the cohort-specific SIIs where the weights are defined by each pairwise complete cases sample size of each cohort. To avoid any disclosure of individual participant data, CHOP cohort is not presented in the panels of externalising problems because there are less than 3 children with positive or negative answers in one maternal educational level for this variable.

**Figure S17:** Prevalence or mean of early-life factors at each level of scaled maternal education using the complete cases samples for **female** children. The slope of each trendline defines the Slope Index of Inequality. To avoid any disclosure of individual participant data, BiB cohort is not presented in the panel of maternal smoking in pregnancy because there are less than 3 children with positive or negative answers in one maternal educational level for this variable.

**Figure S18:** Slope Index of Inequalities between scaled maternal education and early-life risk factors as calculated based on trendlines in Figure S17. The combined SII in each panel is calculated as the weighted average of the cohort-specific SIIs. The weights are defined by each pairwise complete cases sample size of each cohort. To avoid any disclosure of individual participant data, BiB cohort is not presented in the panel of maternal smoking in pregnancy because there are less than 3 children with positive or negative answers in one maternal educational level for this variable.

**Figure S19:** Prevalence of externalising (A) and internalising problems (B) at each level of scaled maternal education using the maternal education and the outcome variables scaled and dichotomised respectively using the sample sizes of the complete cases samples for **female** children. The slope of each trendline defines the Slope Index of Inequality. The forest plots summarise the SIIs and their 95% confidence intervals for externalising (C) and internalising (D) problems. The combined SII is calculated as the weighted average of the cohort-specific SIIs where the weights are defined by each pairwise complete cases sample size of each cohort. To avoid any disclosure of individual participant data, BiB cohort is not presented in the panels of internalising problems because there are less than 3 children with positive or negative answers in one maternal educational level for this variable.

***Robustness tests: Household income as an alternative exposure***

We considered household income at baseline as an alternative exposure in a sensitivity analysis. The household income variable was harmonized across cohorts using the Equivalized Household Income Indicator (EHII) as an indicator of the total disposable monthly household income standardized for the household size and composition. The EHII variable was developed using external data from the 2011 country‐specific European Surveys on Income and Living Condition (EUSILC) and internal data from each cohort (7). The Australian cohort Raine Study is therefore excluded from this sensitivity analysis due to unavailability of such information. We used a harmonised variable that clustered household income in quintiles (thus around 20% of participants of the initial sample of each cohort are included in each quintile). We calculated SIIs using household income as the exposure measure of SECs.

We found fairly consistent results across the participating cohorts when using household income as an alternative exposure of SECs. The baseline characteristics of the participants in the complete cases sample (see flow chart in Figure S20) among children with information on household income are shown in Table S8. The SIIs of mediators, confounder and outcomes are shown in Figures S21-S23. Children leaving in households with income in the lower quintile had a 7.9 and a 6.6 percentage point increased prevalence of externalising and internalising problems respectively compared to children leaving in households with income in the upper quintile. This sensitivity analysis is not fully comparable with our main analysis since household income and maternal education do not cover exactly the same SECs.

**Figure S20:** Flow chart showing how the final size (and the percentage relative to the initial sample) of the complete cases is derived in each cohort based on the availability of measurements for the exposure (household income), outcome and mediators/confounders. The Raine Study is excluded from this sensitivity analysis since EUSILC income data is not available (relevant) for this non-European cohort.

**Table S8:** Baseline characteristics of the participants in the complete cases sample among children with information on household income.

|  | **ALSPAC** | **BiB** | **CHOP** | **DNBC** | **ELFE** | **INMA** | **MoBa** |
| --- | --- | --- | --- | --- | --- | --- | --- |
| **Study population** |  |  |  |  |  |  |  |
| n | 7406 | 454 | 608 | 50420 | 9597 | 731 | 62949 |
| **Maternal age at birth, in years** |  |  |  |  |  |  |  |
| Mean (SD) | 29.0 (4.4) | 28.1 (5.6) | 30.8 (4.6) | 30.1 (4.1) | 30.9 (4.6) | 32.3 (3.8) | 30.5 (4.4) |
| **Household income, quintiles** |  |  |  |  |  |  |  |
| 1 (n, %) | 1146 (15.5%) | 75  (16.5%) | 79  (13.0%) | 9534 (18.9%) | 1253 (13.1%) | 66  (9.0%) | 10858 (17.2%) |
| 2 (n, %) | 1417 (19.1%) | 89  (19.6%) | 100 (16.4%) | 10148 (20.1%) | 1822 (19.0%) | 112 (15.3%) | 12196 (19.4%) |
| 3 (n, %) | 1581 (21.3%) | 104  (22.9%) | 125 (20.6%) | 10196  (20.2%) | 2087  (21.7%) | 155 (21.2%) | 13111 (20.8%) |
| 4 (n, %) | 1608 (21.7%) | 93 (20.5%) | 152 (25.0%) | 10291 (20.4%) | 2159 (22.5%) | 192 (26.3%) | 12834 (20.4%) |
| 5 (n, %) | 1654 (22.3%) | 93 (20.5%) | 152 (25.0%) | 10251 (20.3%) | 2276 (23.7%) | 206 (28.2%) | 13950 (22.2%) |
| **Maternal smoking in pregnancy** |  |  |  |  |  |  |  |
| No (n, %) | 5794 (78.2%) | 412 (90.7%) | 433 (71.2%) | 38408 (76.2%) | 8032 (83.7%) | 553 (75.6%) | 49847 (79.2%) |
| Yes (n, %) | 1612 (21.8%) | 42 (9.3%) | 175 (28.8%) | 12012 (23.8%) | 1565 (16.3%) | 178 (24.4%) | 13102 (20.8%) |
| **Gestational age, in days** |  |  |  |  |  |  |  |
| Mean (SD) | 279.8 (11.6) | 275.9 (12.3) | 282.8  (8.4) | 280.0 (11.3) | 277.7  (9.7) | 279.3  (9.6) | 279.2 (12.4) |
| **Birth weight for gestational age** |  |  |  |  |  |  |  |
| Small (n, %) | 510 (6.9%) | 68 (15.0%) | 53 (8.7%) | 1703 (3.4%) | 620 (6.5%) | 63 (8.6%) | 1742 (2.8%) |
| Normal or Large (n, %) | 6896 (93.1%) | 386  (85.0%) | 555 (91.3%) | 48717 (96.6%) | 8977 (93.5%) | 668  (91.4%) | 61207 (97.2%) |
| **Postpartum depression** |  |  |  |  |  |  |  |
| No (n, %) | 6826 (92.2%) | 360 (79.3%) | 533 (87.7%) | 42796 (84.9%) | 8796 (91.7%) | 650 (88.9%) | 56721 (90.1%) |
| Yes (n, %) | 580 (7.8%) | 94  (20.7%) | 75  (12.3%) | 7624 (15.1%) | 801  (8.3%) | 81  (11.1%) | 6228 (9.9%) |
| **Child ever breast fed** |  |  |  |  |  |  |  |
| No (n, %) | 1693 (22.9%) | 69  (15.2%) | 164 (27.0%) | 779  (1.5%) | 2270 (23.7%) | 56  (7.7%) | 3985 (6.3%) |
| Yes (n, %) | 5713 (77.1%) | 385 (84.8%) | 444 (73.0%) | 49641 (98.5%) | 7327 (76.3%) | 675 (92.3%) | 58964 (93.7%) |
| **Externalising problems** |  |  |  |  |  |  |  |
| Score < the 90^th^ percentile^*^ (n, %) | 6537 (88.3%) | 404 (89.0%) | 548 (90.1%) | 45252 (89.8%) | 8517 (88.7%) | 655 (89.6%) | 56258 (89.4%) |
| Score ≥ the 90^th^ percentile^*^ (n, %) | 869  (11.7%) | 50  (11.0%) | 60  (9.9%) | 5168 (10.2%) | 1080 (11.3%) | 76  (10.4%) | 6691 (10.6%) |
| **Internalising problems** |  |  |  |  |  |  |  |
| Score < the 90^th^ percentile^*^ (n, %) | 6633 (89.6%) | 403 (88.8%) | 547 (90.0%) | 45224 (89.7%) | 8506 (88.6%) | 655 (89.6%) | 54903 (87.2%) |
| Score ≥ the 90^th^ percentile^*^ (n, %) | 773 (10.4%) | 51 (11.2%) | 61 (10.0%) | 5196 (10.3%) | 1091 (11.4%) | 76 (10.4%) | 8046 (12.8%) |

^*^ Using the 90^th^ percentile values of the complete cases sample of each cohort

**Figure S21:** Prevalence or mean of early-life factors at each level of scaled household income used as an alternative exposure measure of SECs. The slope of each trendline defines the Slope Index of Inequality.

**Figure S22:** Slope Index of Inequalities between scaled household income and early-life risk factors as calculated based on trendlines in Figure S21. The combined SII in each panel is calculated as the weighted average of the cohort-specific SIIs. The weights are defined by each pairwise complete cases sample size of each cohort.

**Figure S23:** Prevalence of externalising (A) and internalising problems (B) at each level of scaled household income quintile. The slope of each trendline defines the Slope Index of Inequality. The forest plots summarise the SIIs and their 95% confidence intervals for externalising (C) and internalising (D) problems. The combined SII is calculated as the weighted average of the cohort-specific SIIs where the weights are defined by each pairwise complete cases sample size of each cohort.

**Appendix S3: Supplementary information for cohort-specific statements**

**Box S3: Description of the participating cohorts:**

**ALSPAC:** Pregnant women resident in Avon, UK with expected dates of delivery between 1st April 1991 and 31st December 1992 were invited to take part in the study. 20,248 pregnancies have been identified as being eligible and the initial number of pregnancies enrolled was 14,541. Of the initial pregnancies, there was a total of 14,676 foetuses, resulting in 14,062 live births and 13,988 children who were alive at 1 year of age. When the oldest children were approximately 7 years of age, an attempt was made to bolster the initial sample with eligible cases who had failed to join the study originally. As a result, when considering variables collected from the age of seven onwards (and potentially abstracted from obstetric notes) there are data available for more than the 14,541 pregnancies mentioned above: The number of new pregnancies not in the initial sample (known as Phase I enrolment) that are currently represented in the released data and reflecting enrolment status at the age of 24 is 906, resulting in an additional 913 children being enrolled (456, 262 and 195 recruited during Phases II, III and IV respectively). The phases of enrolment are described in more detail in the cohort profile paper and its update (see footnote 5 below). The total sample size for analyses using any data collected after the age of seven is therefore 15,447 pregnancies, resulting in 15,658 foetuses. Of these 14,901 children were alive at 1 year of age. Of the original 14,541 initial pregnancies, 338 were from a woman who had already enrolled with a previous pregnancy, meaning 14,203 unique mothers were initially enrolled in the study. As a result of the additional phases of recruitment, a further 630 women who did not enrol originally have provided data since their child was 7 years of age. This provides a total of 14,833 unique women (G0 mothers) enrolled in ALSPAC as of September 2021. G0 partners were invited to complete questionnaires by the mothers at the start of the study and they were not formally enrolled at that time. 12,113 G0 partners have been in contact with the study by providing data and/or formally enrolling when this started in 2010. 3,807 G0 partners are currently enrolled. Additional information can be found in <https://www.bristol.ac.uk/alspac/>. Please note that the study website contains details of all the data that is available through a fully searchable data dictionary and variable search tool: <http://www.bristol.ac.uk/alspac/researchers/our-data/>.

**BiB:** Established in 2007, the Born in Bradford cohort study, also known as BiB, aims to investigate the impact of genetic, nutritional, environmental, behavioural, and social factors on health and development in a deprived multi-ethnic population, both during childhood and later in adult life. Bradford, home to one of the busiest maternity units in the UK, situated at Bradford Royal Infirmary, witnesses over 6000 new births annually. Women scheduled for delivery at this facility are offered an oral glucose tolerance test (OGTT) between 26 and 28 weeks of gestation, with approximately 80% opting for the test. Women are informed about the study during their booking appointment with a midwife and are invited to participate. Upon attendance at the OGTT clinic, women provide full consent for recruitment into BiB and complete an interviewer-administered questionnaire. Between March 2007 and November 2010, more than 80% of the women attending the OGTT accepted the invitation, resulting in the recruitment of 12,453 women with 13,776 pregnancies to the cohort. OGTT results were available for 11,442 of them. Partners of the recruited women were also invited to participate in the study. Out of the total 13,776 pregnancies, 13,740 ended in live births. Additional information can be found in <https://borninbradford.nhs.uk>.

**CHOP:** The EU Childhood Obesity Programme established to perform a multicenter intervention trial on new-born infants, to investigate whether feeding infant formulae, which differ in their level of milk proteins, can influence the risk of later childhood obesity. The trial took place in five European countries from where the data were collected: Belgium, Germany, Italy, Poland and Spain. Additional information can be found in <http://www.metabolic-programming.org/obesity/>

**DNBC:** The Danish National Birth Cohort established to investigate the causal link between exposures in early life and disease later on and the possibilities for disease prevention. In total, 100,407 pregnancies with a median gestational age of 10 weeks were enrolled across Denmark. From these pregnancies, 96,822 participants were born alive from 1996 to 2003 by 88,122 unique mothers. Additional information can be found in <https://www.dnbc.dk>.

**ELFE:** ELFE, has a primary focus on investigating the impact of various exposures on health outcomes, aims to shed light on the intricate interplay between familial history, socio-economic conditions, education, and a range of environmental stressors. These stressors encompass heavy metals, organic volatile compounds, pesticides, persistent organic pollutants (POP), phthalates, environmental tobacco smoke (ETS), molds, air pollutants, electromagnetic fields (EMF), ultraviolet (UV) radiation, ionizing radiations, and noise. The study seeks to evaluate outcomes related to neurodevelopment and school achievements, fertility, puberty and sexual development, asthma and allergies, obesity and growth, as well as hearing loss. The cohort comprises 20,000 children which is approximately 3% of the nation-based source population. The enrolment period spans from April 1, 2011, to December 5, 2011, with enrolment occurring at birth. Enrolment criteria specify that participants must have a recorded date of delivery, major mothers, term pregnancies of 33 weeks of amenorrhea or more, and single or twin pregnancies. Maternal mental incapacity serves as the sole exclusion criterion. The planned age for children at the end of the follow-up is 20 years, aiming to track and analyse their health outcomes over an extended period. Additional information can be found in <https://www.elfe-france.fr/en>.

**INMA:** The INMA (Environment and Childhood) Project is a network of birth cohorts in Spain focused on studying the impact of environmental pollutants in air, water, and diet during pregnancy and early childhood on child growth and development. The project includes pregnant women residing in specific study areas [Ribera d’Ebre, Menorca, Granada, Valencia, Sabadell, Asturias, and Gipuzkoa] and their children. Inclusion criteria for mothers were residence in a study area, being over 16 years old, a singleton pregnancy, no assisted reproduction treatment, a desire to deliver in the reference hospital, and no communication problems. Each cohort had a unique recruitment period. In Ribera d’Ebre and Granada, mothers were recruited during hospital admission for delivery between March 1997 and December 1999 and October 2000 and July 2002, respectively. Menorca invited eligible pregnant women presenting for prenatal care over 12 months starting in mid-1997. Valencia, Sabadell, Asturias, and Gipuzkoa recruited during the first prenatal visit (10–13 weeks of gestation) in the main public hospital or health center. Recruitment periods varied across areas, ranging from November 2003 to January 2008. The participation rate varied from 45% to 98%, with 96% in Ribera d’Ebre, 98% in Menorca, 54% in Valencia, 60% in Sabadell, 45% in Asturias, and 68% in Gipuzkoa. Reasons for not participating in Sabadell and Gipuzkoa included 27.6% not wanting to participate, 30.3% citing lack of time, 9.3% expressing no interest, and 32.7% could not be located for the baseline interview after being identified as eligible by their attending physician. In total, 3,174 women were recruited during pregnancy, with 2,998 mother-child pairs followed up at birth. Additional information can be found in <https://www.proyectoinma.org/>.

**MoBa:** The Norwegian Mother, Father and Child Cohort Study (MoBa) MoBa is a Norwegian population-based longitudinal cohort study of around 114,500 children, 95,000 mothers and 75,000 fathers. Recruitment of pregnant women attending routine ultrasound examination took place between 1999 and 2009, and MoBa has since grown to become one of the largest population studies in the world. Baseline questionnaires were completed at around 15 weeks’ gestation, and two further prenatal questionnaires were administered at around 17-22 and 30 weeks’ gestation. In addition, fathers completed a questionnaire at around 15 weeks’ gestation. Follow-up questionnaires after birth were completed at 6 months, 18 months, 36 months, 5 years, 7 years, and 8 years of age, and data are currently being collected through questionnaires for children aged 13, 14 and 18 years. The full cohort is being genotyped, with more than 230,000 participants (including 70,000 trios) genotyped to date, and whole genome methylation data available on around 15,000 participants. In addition, the cohort recently launched the release of other genetic, metabolomic and proteomic biomarker data. The purpose of MoBa is to improve our knowledge of diseases and health, by estimating exposure-outcome associations among children and their parents from preconception and pregnancy throughout life. Additional information can be found in <https://www.fhi.no/en/studies/moba/>.

**The Raine Study:** The Raine Study began in 1989 in Perth in Western Australia. It is a multigenerational longitudinal research study following a cohort of ‘Western Australians’. Initially, 2,900 pregnant women recruited at 18 weeks of gestation. Additional information can be found in <https://rainestudy.org.au>.

**Table S9:** Informed consent statements

| **Cohort** | **Statement** |
| --- | --- |
| ALSPAC | Informed consent for the use of data collected via questionnaires and clinics was obtained from participants following the recommendations of the ALSPAC Ethics and Law Committee at the time. Participants can contact the study team at any time to retrospectively withdraw consent for their data to be used. Study participation is voluntary and during all data collection sweeps, information was provided on the intended use of data. |
| BiB | All participants gave written informed consent. |
| CHOP | Written informed consent was obtained from all parents before enrolment and at 6 and 11 years. At 8 and 11 years also children consent was obtained. |
| DNBC | Informed consent was obtained from participants upon enrolment |
| ELFE | Informed consent was signed by the parents or the mother alone, with the father being informed of his right to deny consent for participation. |
| INMA | All participants provided written informed consent before enrolment to the study: All participants, regardless of the study area, are informed of the aims of the study. They are given an information sheet and a paper consent form by post. A telephone call is made to ask if they have received the information sheet, and an appointment is made on the same call. They bring the signed consent form to the appointment. |
| MoBa | MoBa is conducted according to the guidelines laid down in the declaration of Helsinki, and written informed consent was obtained from all participants. A detailed protocol of the study including the consent can be found elsewhere (http://www.fhi.no/morogbarn). |
| The Raine Study | Parental or guardian consent for child participation in the Raine Study was obtained at pregnancy, birth, and at ages 1, 2, 3, 5, 8, 10, 14, and 17 years; participant assent was obtained at ages 14 and 17. |

**Table S10:** Ethics approval statements

| **Cohort** | **Statement** |
| --- | --- |
| ALSPAC | Ethical approval for the study was obtained from the ALSPAC Ethics and Law Committee and the Local Research Ethics Committees |
| BiB | Ethics approval has been obtained for the main platform study and all of the individual substudies from the Bradford Research Ethics Committee |
| CHOP | The study was approved by the local ethics committees of all study centers |
| DNBC | The DNBC complies with the Declaration of Helsinki and was approved by the Danish National Committee on Biomedical Research Ethics |
| ELFE | Ethical approvals for data collection in maternity units and for each data collection wave during follow-up were obtained from the national advisory committee on information processing in health research (CCTIRS: Comité Consultatif sur le Traitement de l’Information en matière de Recherche dans le domaine de la Santé), the national data protection authority (CNIL: Comission Nationale Informatique et Liberté) and, in case of invasive data collection such as biological sampling, the committee for protection of persons engaged in research (CPP: Comité de Protection des Personnes). The ELFE study was also approved by the national committee for statistical information (CNIS: Conseil National de l’Information Statistique). |
| INMA | The INMA project was approved by the ethics committee in each area. |
| MoBa | The establishment and data collection in MoBa was previously based on a license from the Norwegian Data protection agency and approval from The Regional Committee for Medical Research Ethics, and it is now based on regulations related to the Norwegian Health Registry Act. |
| The Raine Study | The Raine Study dataset is governed by a consolidated ethics approval from the University of Western Australia Human Research Ethics Committee (Approval No. RA/4/20/5722; granted on 29 April 2020). |

**Table S11:** Funding and acknowledgement statements

| **Cohort** | **Statement** |
| --- | --- |
| ALSPAC | We are extremely grateful to all the families who took part in this study, the midwives for their help in recruiting them, and the whole ALSPAC team, which includes interviewers, computer and laboratory technicians, clerical workers, research scientists, volunteers, managers, receptionists and nurses.  The UK Medical Research Council and Wellcome (Grant ref: 217065/Z/19/Z) and the University of Bristol provide core support for ALSPAC. This publication is the work of the authors and will serve as guarantors for the contents of this paper. |
| BiB | The authors acknowledge that Born in Bradford is only possible because of the enthusiasm and commitment of the children and parents in Born in Bradford. We are grateful to all participants, health professionals and researchers who have made Born in Bradford happen.  Born in Bradford receives funding from by a joint grant from the UK Medical Research Council (MRC) and UK Economic and Social Science Research Council (ESRC) [MR/N024391/1]; the British Heart Foundation [CS/16/4/32482]; a Wellcome Infrastructure Grant [WT101597MA]; The National Institute for Health Research under its Applied Research Collaboration for Yorkshire and Humber [NIHR200166]. The views expressed are those of the author(s), and not necessarily those of the NHS, the NIHR or the Department of Health and Social Care. |
| CHOP | The CHOP study has been carried out with partial financial support from the Commission of the European Community, specific RTD Programme "Quality of Life and Management of Living Resources", within the Fifth Framework Program (research grants no. QLRT-2001-00389, QLAM 2001-00582 and QLK1-CT-200230582), the Sixth Framework Program (contract no. 007036), and Seventh Framework Programme (EarlyNutrition; grant agreement no. 289346), the EU H2020 project LIFECYCLE under grant no. 733206 and the European Research Council Advanced Grant META-GROWTH (ERC-2012-AdG – no.322605) and with financial support from Polish Ministry of Science and Higher Education (2571/7.PR/2012/2). This project has received funding from the European Union’s Horizon 2020 research and innovation programme under the ERA-NET Cofund action N° 727565 and from the German Federal Ministry of Education and Research, Berlin, N° 01EA2101 (EndObesity) under the umbrella of the European Joint Programming Initiative “A Healthy Diet for a Healthy Life” (JPI HDHL), and from the German Federal Ministry of Education and Research, Berlin, N° 01EA2203A (BiomarKid) under the umbrella of the European Joint Programming Initiative “A Healthy Diet for a Healthy Life” (JPI HDHL) and of the ERA-NET Cofund ERA-HDHL (Ga N° 696295 of the EU Horizon 2020 Research and Innovation Programme). This work was co-funded by the German Ministry of Education and Research as part of the German Center for Child and Adolescent Health (DZKJ), 01GL2406A. This article does not necessarily reflect the views of the Commission and in no way anticipates the future policy in this area. No funding bodies had any role in the study design, data collection and analysis, decision to publish, or preparation of the manuscript.  The authors would particularly like to thank all the cohort participants for their generous collaboration. Furthermore, thanks to all persons who designed and conducted the study, entered the data, and participated in the data analysis and who are represented by the European Childhood Obesity Trial Study Group participants: B Koletzko, V Grote, M Totzauer, K Guerlich, P Schwarzfischer, N Aumüller, V Luque, M Zaragoza-Jordana, N Ferré, J Escribano, R Closa-Monasterolo, A Xhonneux, JP Langhendries, E Verduci, E Riva, D Gruszfeld. |
| DNBC | The Danish National Birth Cohort was established with a significant grant from the Danish National Research Foundation. Additional support was obtained from the Danish Regional Committees, the Pharmacy Foundation, the Egmont Foundation, the March of Dimes Birth Defects Foundation, the Health Foundation and other minor grants. The DNBC Biobank has been supported by the Novo Nordisk Foundation and the Lundbeck Foundation. Follow‐up of mothers and children have been supported by the Danish Medical Research Council (SSVF 0646, 271‐08‐0839/06‐066023, O602‐01042B, 0602‐02738B), the Lundbeck Foundation (195/04, R100‐A9193), The Innovation Fund Denmark 0603‐00294B (09‐067124), the Nordea Foundation (02‐2013‐2014), Aarhus Ideas (AU R9‐A959‐13‐S804), University of Copenhagen Strategic Grant (IFSV 2012), and the Danish Council for Independent Research (DFF – 4183‐00594 and DFF ‐ 4183‐00152). The authors would like to thank the data donors, the cohort’s founding fathers, the scientific managerial team, and DNBC secretariat for being, establishing, developing and consolidating the Danish National Birth Cohort. |
| ELFE | The authors are grateful to 1) the former members of the Elfe unit without whom the project would never have started 2) the expertise and assistance of members of the unit for support functions, 3) all the researchers who contribute to the projects as members of the Elfe thematic groups and especially their coordinators; 4) all the field research assistants and interviewers; 5) and above all, all the Elfe families who have placed their confidence in us and given up their time to the study. |
| INMA | The authors acknowledge support from the European Union’s Horizon 2020 research and innovation programme-EU.3.1.2. (874583–ATHLETE project); grants from UE (FP7-ENV-2011 cod 282957 and HEALTH.2010.2.4.5-1), Spain: ISCIII (Red INMA G03/176, CB06/02/0041; FIS-FEDER: PI03/1615, PI04/1509, PI04/1112, PI04/1931, PI05/1079, PI05/1052, PI06/1213, (FIS-PI06/0867, FIS-PI09/00090, FIS-PI13/02187, FIS-PI18/01142, FIS-PI18/01237 incl FEDER funds) PI07/0314, PI09/02647, PI11/01007, PI11/02591, PI11/02038, PI12/00610, PI13/1944, PI13/2032, PI14/00891, PI14/01687, PI16/1288, PI17/00663, PI16/1288 and PI1338; Department of Health of the Basque Government (2005111093, 2009111069, 2013111089, 2015111065 and 2018111086); the Provincial Government of Gipuzkoa (DFG06/002, DFG08/001, DFG15/221 and DFG 89/17); grants from Instituto de Salud Carlos III (Red INMA G03/176; CB06/02/0041; PI041436; PI081151 incl. FEDER funds), Generalitat de Catalunya-CIRIT 1999SGR 00241, Fundació La marató de TV3 (090430); grants from Instituto de Salud Carlos III (PI06/0867, PI11/00610, FIS-PI09/00090, FIS-PI13/02187, FIS-PI13/02406, and FIS-PI18/01142 incl. FEDER funds) and annual agreements with the municipalities of the study area (Zumarraga, Urretxu, Legazpi, Azkoitia, Azpeitia and Beasain).  The authors acknowledge all volunteers participating in the study, and interviewers and psychologists. |
| MoBa | This work was partly supported by the Research Council of Norway through its Centres of Excellence funding scheme, project number 262700. The authors are grateful to all the participating families in Norway who take part in this on-going cohort study. |
| The Raine Study | We gratefully acknowledge all Raine Study participants and their families for their continued participation in the study, as well as the Raine Study team for study co-ordination and data collection. We also thank the NHMRC and the Raine Medical Research Foundation for their support. The core management of the Raine Study is funded by The University of Western Australia, Curtin University, The Kids Research Institute Australia, Women and Infants Research Foundation, Edith Cowan University, Murdoch University, The University of Notre Dame Australia and the Western Australian Future Health Research and Innovation Fund (2023-2024; Grant ID WACSOSP2023-2024). The Raine Study Gen2-14 year follow-up was funded by NHMRC Grant ID 1021105 and the Raine Study Gen2-17 year follow-up was funded by NRMRC Grant ID 353514. |

# **References**

| 1. | Goodman R. The Strengths and Difficulties Questionnaire: A Research Note. Journal of Child Psychology and Psychiatry. 1997;: 581-586. |
| --- | --- |
| 2. | Achenbach T, Edelbrock C. Manual for the Child Behavior Checklist. Burlington, VT:; 1991. |
| 3. | Ebesutani C, Bernstein A, Martinez JI, Chorpita BF, Weisz JR. The Youth Self Report: Applicability and Validity Across Younger and Older Youths. Journal of Clinical Child & Adolescent Psychology. 2011; 40(2): 338-346. |
| 4. | Villar J, Giuliani F, Fenton T, Ohuma E, Ismail L, Kennedy S, et al. INTERGROWTH-21st very preterm size at birth reference charts. Lancet. 2016; 387(10021): 844-845. |
| 5. | Hutcheon JA, Jacobsen GW, Kramer MS, Martinussen M, Platt RW. Small Size at Birth or Abnormal Intrauterine Growth Trajectory: Which Matters More for Child Growth? American Journal of Epidemiology. 2016; 183(12): 1107–1113. |
| 6. | Van Buuren S, Groothuis-Oudshoorn K. mice: Multivariate Imputation by Chained Equations in R. Journal of Statistical Software. 2011; 45(3): 1-67. |
| 7. | Pizzi C, Richiardi M, Charles MA, Heude B, Lanoe JL, Lioret S, et al. Measuring Child Socio-Economic Position in Birth Cohort Research: The Development of a Novel Standardized Household Income Indicator. International Journal of Environmental Research and Public Health. 2020; 17(5). |
